# Population Health Management and Guideline-Concordant Care in CKD: A Secondary Analysis of Kidney Coordinated HeAlth Management Partnership

**DOI:** 10.1681/ASN.0000000544

**Published:** 2024-11-01

**Authors:** Melanie R. Weltman, Linda-Marie U. Lavenburg, Zhuoheng Han, Alaa A. Alghwiri, Mitra Mosslemi, Bruce L. Rollman, Gary S. Fischer, Thomas D. Nolin, Jonathan G. Yabes, Manisha Jhamb

**Affiliations:** 1 Renal-Electrolyte Division, Department of Medicine, University of Pittsburgh School of Medicine, Pittsburgh, Pennsylvania; 2 Department of Pharmacy and Therapeutics, University of Pittsburgh School of Pharmacy, Pittsburgh, Pennsylvania; 3 Department of Epidemiology, University of Pittsburgh School of Public Health, Pittsburgh, Pennsylvania; 4 Division of General Internal Medicine, Department of Medicine, University of Pittsburgh, Pittsburgh, Pennsylvania; 5 Center for Behavioral Health, Media, and Technology, University of Pittsburgh School of Medicine, Pittsburgh, Pennsylvania; 6 Division of General Internal Medicine, Department of Medicine and Biostatistics, Center for Research on Heath Care, University of Pittsburgh, Pittsburgh, Pennsylvania

**Keywords:** BP, clinical trial, diabetes mellitus, randomized controlled trials

## Abstract

**Key Points:**

- Implementation gaps in guideline-concordant care for CKD are associated with poor clinical outcomes.
- A population health management–based, multidisciplinary approach improved exposure days to sodium-glucose cotransporter-2 inhibitor and glucagon-like peptide-1 receptor agonists compared with usual care.
- Angiotensin-converting enzyme inhibitor/angiotensin receptor blocker in albuminuric patients and statin use was not improved, nor was BP control, glycemic control, or albuminuria testing.

**Background:**

Gaps in guideline-concordant care for CKD lead to poor outcomes. The Kidney Coordinated HeAlth Management Partnership (K-CHAMP) cluster randomized trial tested the effect of a population health management intervention versus usual care on CKD progression and evidence-based care delivery in the primary care setting.

**Methods:**

K-CHAMP included adults aged 18–85 years with eGFR<60 ml/min per 1.73 m^2^ and moderate-high risk of CKD progression who were not seeing a nephrologist. The multifaceted intervention included nephrology e-consult, pharmacist-led medication management, and patient education. In this *post hoc* analysis, we evaluate the effectiveness of K-CHAMP on guideline-concordant care processes (BP and glycemic control, annual albuminuria testing) and medication exposure days (angiotensin-converting enzyme inhibitor [ACEi]/angiotensin receptor blocker [ARB], moderate-high intensity statin, sodium-glucose cotransporter-2 inhibitor [SGLT2i], glucagon-like peptide-1 receptor agonists [GLP-1RA]). Given multiplicity of outcomes, Benjamini–Hochberg method was used to control false discovery rate.

**Results:**

All 1596 (754 intervention, 842 usual care) enrolled patients (mean age 74±9 years, eGFR 37±8 ml/min per 1.73 m^2^, 928 [58%] female, 127 [8%] Black) were analyzed. After a median 17-month follow-up, intervention arm patients had significantly higher exposure days per year to SGLT2i (56 versus 32 days; relative benefit 1.72; 95% confidence interval [CI], 1.14 to 2.30) and GLP-1RA (78 versus 29 days; relative benefit 2.65; 95% CI, 1.59 to 3.71) compared with usual care in adjusted analysis. At study initiation in 2019, similar proportion of patients were prescribed SGLT2i and/or GLP-1RA in intervention and control arm (8% versus 6%, respectively; rate ratio 1.23; 95% CI, 0 to 2.99), but by 2022, prescription of these medications was significantly higher in intervention arm (44% versus 27%, respectively; rate ratio 1.63; 95% CI, 1.32 to 1.94). There was no significant difference in any process measures or exposure days to ACEi/ARB in patients with albuminuria or moderate-high intensity statin.

**Conclusions:**

K-CHAMP was effective in accelerating implementation of SGLT2i and GLP-1RA but did not increase ACEi/ARB in patients with albuminuria or moderate-high intensity statin use or improve BP control, glycemic control, or albuminuria testing in individuals with CKD in the primary care setting.

**Clinical Trial registry name and registration number:**

K-CHAMP, NCT03832595.

## Visual Abstract

**Figure:**
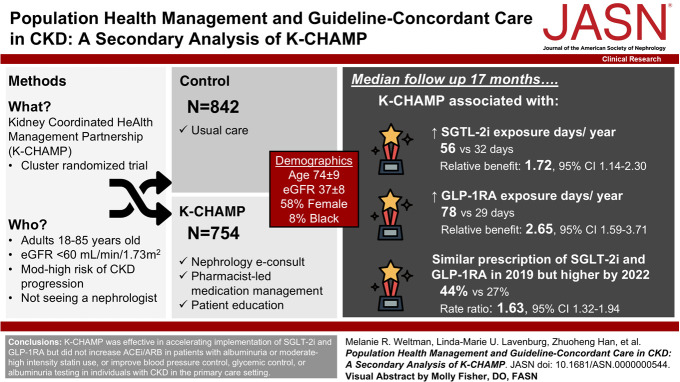

## Introduction

CKD has a high prevalence in the United States, affecting 14% of all adults,^1^ and is associated with high morbidity, mortality, and health care costs.^2–4^ CKD-associated mortality has increased over the last two decades, both globally^5^ and in the United States,^3,4^ highlighting CKD as a pressing public health problem. Large-scale clinical studies indicate that despite availability of evidence-based clinical practice guidelines, gaps in CKD guideline-concordant care have persisted across all racial and ethnic groups over the years.^6–9^ These gaps include inadequate hypertension and diabetes control and suboptimal use of statins and angiotensin-converting enzyme inhibitors (ACEi)/angiotensin receptor blockers (ARB).^6,9–12^ Similarly, use of new guideline-recommended kidney protective medications including sodium-glucose cotransporter-2 inhibitors (SGLT2i), glucagon-like peptide-1 receptor agonists (GLP-1RA), and nonsteroidal mineralocorticoid receptor antagonist is suboptimal in CKD.^7,13^ These gaps in guideline-concordant care have the potential to widen because of the growing CKD population and increasing burden on primary care to manage these highly complex patients.^14^ There is substantial opportunity and unmet need for improving CKD care delivery, especially with the advent of new CKD-modifying therapies.

Our team developed and evaluated effectiveness of an electronic health record (EHR)-based population health management approach, the Kidney Coordinated HeAlth Management Partnership (K-CHAMP), to improve CKD outcomes. This multifaceted intervention addressed barriers at the primary care provider (PCP), patient, and health system levels for comanagement of patients with CKD. Over a median follow-up of 17 months, there was no significant difference in rate of the primary outcome—time to ≥40% reduction in eGFR or kidney failure (adjusted hazards ratio, 0.96; 95% confidence interval [CI], 0.67 to 1.38; *P* = 0.82)—between the intervention and usual care arms.^15^ This secondary data analysis of the K-CHAMP trial evaluates the effect of the K-CHAMP intervention on guideline-concordant care process measures (hypertension and diabetes control, annual albuminuria testing), medication prescription (ACEi/ARB, moderate-high intensity statin, SGLT2i and GLP-1RA), and temporal trends in the medications use during study period (May 2019–July 2022).

## Methods

### Study Design

K-CHAMP design and primary outcome results were previously published.^15,16^ Briefly, K-CHAMP was a clustered randomized trial that compared the effectiveness of an EHR-based, population health management intervention with usual care on CKD progression in 1596 participants from 101 PCP practices located across western Pennsylvania. The study protocol was approved by the Institutional Review Board and the Quality Improvement Committee at the University of Pittsburgh.

### Study Population

K-CHAMP included patients aged 18–85 years, with eGFR <60 ml/min per 1.73 m^2^, at high risk of CKD progression, not seeing a nephrologist. High-risk CKD was eGFR 15–29 ml/min per 1.73 m^2^, 5-year risk of kidney failure ≥4% using validated four-variable kidney failure risk equation,^17^ or based on an internal machine learning–based risk prediction model.^16^ Patients with baseline eGFR <15 ml/min per 1.73 m^2^, receiving maintenance dialysis, or had history of kidney transplant were excluded. The current secondary analyses are restricted to relevant subcohorts (Table 1).

**Table 1:** Reference and definition of guideline-concordant care indicators

Metric	Guideline Recommendation (Strength/Grade for Guidelines)^a^	Guideline-Concordant Care Definition (Numerator^a^)	Cohort Evaluated (Denominator)
**Process outcomes**			
BP control	ACC/AHA 2017^18^ Adults with hypertension and CKD should be treated to a BP goal of <130/80 mm Hg (class 1, level B–R^SR^ [for systolic BP], level C–EO [for diastolic BP]) KDIGO 2012^19^ We recommend that in both diabetic and nondiabetic adults with CKD and urine albumin excretion <30 mg/24 h (or equivalent) whose office BP is consistently >140 mm Hg systolic or >90 mm Hg diastolic be treated with BP-lowering drugs to maintain a BP that is consistently ≤140 mm Hg systolic and ≤90 mm Hg diastolic (level 1, grade B) We suggest that in both diabetic and nondiabetic adults with CKD and with urine albumin excretion of ≥30 mg/24 h (or equivalent) whose office BP is consistently >130 mm Hg systolic or >80 mm Hg diastolic be treated with BP-lowering drugs to maintain a BP that is consistently ≤130 mm Hg systolic and ≤80 mm Hg diastolic (level 2, grade D) KDIGO 2021^20^ We suggest that adults with high BP and CKD be treated with a target systolic BP of <120 mm Hg, when tolerated, using standardized office BP measurement (level 2, grade B)	Patients with outpatient systolic BP <130 mm Hg and diastolic BP <80 mm Hg	All included patients
Annual UACR	KDIGO 2012^19^ Assess GFR and albuminuria at least annually in people with CKD. Assess GFR and albuminuria more often for individuals at higher risk of progression and/or when measurement will impact therapeutic decisions (not graded)	Patients with UACR measurement ≥1 time in last 1 yr	All included patients
Glycemic control	KDIGO 2020^21^ We recommend an individualized HbA1c target ranging from <6.5% to <8.0% in patients with diabetes and CKD not treated with dialysis (level 1, grade C) ADA 2019^22^ A reasonable HbA1c goal for many nonpregnant adults is <7% (53 mmol/mol) (level of evidence A)	Patients with diabetes with HbA1c <7%	All patients with diabetes (type 1 and type 2)

Metric	Guideline Recommendation	Guideline-Concordant Care Definition (Numerator^a^)	Cohort Evaluated (Denominator)
**Medications**			
ACEi or ARB	KDIGO 2012^19^ We suggest that an ARB or ACEi be used in diabetic adults with CKD and urine albumin excretion 30–300 mg/24 h (or equivalent) (2D) 3.1.7: we recommend that an ARB or ACEi be used in both diabetic and nondiabetic adults with CKD and urine albumin excretion >300 mg/24 h (or equivalent) (level 1, grade B) KDIGO 2021^20^ We recommend starting ACEi or ARB for people with high BP, CKD, and severely increased albuminuria (G1–G4, A3) without diabetes (level 1, grade B) We suggest starting ACEi or ARB for people with high BP, CKD, and moderately increased albuminuria (G1–G4, A2) without diabetes (level 2, grade C) We recommend starting ACEi or ARB for people with high BP, CKD, and moderately to severely increased albuminuria (G1–G4, A2 and A3) with diabetes (level 1, grade B) ACC/AHA 2017^18^ In adults with hypertension and CKD (stage 3 or higher or stage 1 or 2 with albuminuria [≥300 mg/d, or ≥300 mg/g UACR or the equivalent in the first morning void]), treatment with an ACEi is reasonable to slow kidney disease progression (class 2a, level B–R) In adults with hypertension and CKD (stage 3 or higher or stage 1 or 2 with albuminuria [≥300 mg/d, or ≥300 mg/g UACR in the first morning void]), treatment with an ARB may be reasonable if an ACEi is not tolerated (class 2b, level C–EO)	ACEi or ARB exposure days	Patients with UACR >300; with diabetes and UACR ≥30 to ≤300; or with hypertension and UACR ≥30 to ≤300 mg/g at baseline
Moderate-intensity or high-intensity statin	KDIGO 2013^49^ In adults aged 50 yr or older with eGFR <60 ml/min per 1.73 m^2^ but not treated with chronic dialysis or kidney transplantation (GFR categories G3a–G5), we recommend treatment with a statin or statin/ezetimibe combination (grade 1A) In adults aged 18–49 yr with CKD but not treated with long-term dialysis or kidney transplantation, we suggest statin treatment in people with one or more of the following (level 2, grade A) • Known coronary disease (myocardial infarction or coronary revascularization) • Diabetes mellitus • Prior ischemic stroke • Estimated 10-yr incidence of coronary death or nonfatal myocardial infarction >10% ACC/AHA 2018^28^ In adults aged 40–75 yr with LDL-C 70–189 mg/dl (1.7–4.8 mmol/L) who are at 10-yr atherosclerotic cardiovascular disease risk of 7.5% or higher, CKD not treated with dialysis or kidney transplantation is a risk-enhancing factor and initiation of a moderate-intensity statin or moderate-intensity statins combined with ezetimibe can be useful (class 2a, level B–R) In patients who are 75 yr of age or younger with clinical atherosclerotic cardiovascular disease, high-intensity statin therapy should be initiated or continued with the aim of achieving a 50% or greater reduction in LDL-C levels (class 1, level A) In patients 20–75 yr of age with an LDL-C level of 190 mg/dl or higher (4.9 mmol/L), maximally tolerated statin therapy is recommended (class 1, level B–R)	Moderate-intensity or high-intensity statin exposure days	Patients aged 40–75 yr or younger at baseline
SGLT2i	KDIGO 2020^21^ We recommend treating patients with type 2 diabetes, CKD, and an eGFR ≥30 ml/min per 1.73 m^2^ with an SGLT2i (level 1, grade A)	SGLT2i exposure days	Patients with type 2 diabetes and eGFR ≥30 ml/min per 1.73 m^2^ at baseline
GLP-1RA	KDIGO 2020^21^ In patients with type 2 diabetes and CKD who have not achieved individualized glycemic targets, despite use of metformin and SGLT2i treatment, or who are unable to use those medications, we recommend a long-acting GLP-1RA (level 1, grade B)	GLP-1RA exposure days	Patients with type 2 diabetes

Nomenclature for rating recommendations is per cited guidelines. ACC/AHA, American College of Cardiology/American Heart Association; ACEi, angiotensin-converting enzyme inhibitor; ADA, American Diabetes Association; ARB, angiotensin receptor blocker; EO, expert opinion; GLP-1RA, glucagon-like peptide-1 receptor agonist; HbA1c, hemoglobin A1c; KDIGO, Kidney Disease Improving Global Outcomes; LDL-C, LDL cholesterol; R, randomized; R^SR^, randomized (systematic review); SGLT2i, sodium-glucose cotransporter-2 inhibitor; UACR, urine albumin-to-creatinine ratio.

a For numerator, patients are selected from the denominator population.

### Study Intervention and Follow-Up

PCP practices were randomized 1:1 into K-CHAMP intervention versus usual care, and patients were enrolled using an opt-out approach. The intervention bundle included nephrology guidance to the PCP through targeted automated e-consults, pharmacist-led medication management, nurse-delivered patient education, and academic detailing for PCPs. Patients were followed every 4–6 months. Implementation of recommendations, such as ordering of medications and laboratory tests, was at the discretion of PCPs.

Recommendations for use of newer therapeutics were incorporated from 2020 onwards as evidence-based guidelines emerged (Table 1).^21,23^ In accordance with national trends, medication cost, preauthorization requirements, and drug shortages inhibited simultaneous use of both SGLT2i and GLP-1RA in our population.^24,25^ Therefore, recommendations were individualized based on underlying comorbidities.^26^ Moreover, owing to lack of data on benefits and safety of newer therapeutics in older adults with CKD, we did not strongly recommend these in patients older than 75 years and advised use based on shared clinical decision making. Similarly, we recommended PCPs to individualize glycemic and BP targets, especially for older patients, as appropriate.^21^

Owing to coronavirus disease 2019 pandemic–related delays, the enrollment period was extended, resulting in subsequent shortening of the planned follow-up duration from 24 months to until the primary outcome was achieved or the end of intervention period (July 31, 2022).

### Study Outcomes

Guideline-concordant process measures included achievement of BP and glycemic control at end of follow-up and annual albuminuria testing (Table 1). Guideline-concordant medications of interest were ACEi/ARB in patients with albuminuria, moderate-high intensity statin, SGLT2i, and GLP-1RA. Owing to changing guidelines and individualized recommendations described previously, combined exposure days to either SGLT2i or GLP-1RA, or both agents (*i.e*., SGLT2i and/or GLP-1RA) were analyzed in patients with type 2 diabetes. It is important to note that the outcomes for this secondary analysis were receipt of guideline-concordant care, and thus, the definition of outcomes and the subcohorts for evaluation of each outcome are different from those included in our previously published primary outcome article.^15^

Demographic data, including race and ethnicity, were collected from the EHR. Baseline and follow-up BP values were determined by the average of the two nearest outpatient BP measurements (as opposed to average of all BPs during the study period, which was previously published)^15^ before the enrollment date and last study time point, respectively. Hemoglobin A1c (HbA1c) was the most recent value before the enrollment date and last study time point. Urine albumin-creatinine ratio (UACR) was calculated using urine albumin or protein quantification or estimated using urine dipstick protein.^27^ For patients with a UACR result within 1 year before enrollment, albuminuria testing was considered guideline-concordant if a UACR test was repeated within 1 year of baseline UACR test. For patients with no UACR result within 1 year before enrollment, guideline-concordant albuminuria testing was a UACR test completed within 1 year after enrollment date or at least two UACR tests completed less than 1 year apart during follow-up. Patients with less than 1 year of follow-up were excluded from the UACR analysis.

Medication prescription was determined from EHR-level medication order data. Medication exposure days were duration from medication order start date to end date or last study time point, whichever came first. For medication orders with missing end dates and no subsequent start date for the same medication within 1 year, we assumed a 1-year end date, consistent with Pennsylvania Code Title 49, 27.18(i), which allows for prescriptions to be refilled for up to 1 year if authorized by the prescriber. This assumption was applied across all medication categories, with the understanding that long-term therapies for chronic conditions are unlikely to involve short-term supplies without refills. Moderate-intensity and high-intensity statin was defined using the American College of Cardiology/American Heart Association classification.^28^ Angiotensin receptor/neprilysin inhibitor medication class was included under ARB. Notably, guidelines incorporating nonsteroidal mineralocorticoid receptor antagonist use were not released until after the study period; thus, it was not included in this analysis.

### Statistical Analysis

We calculated the proportion of patients receiving guideline-concordant care, along with pointwise 95% CIs, for each calendar year of the study, stratified by intervention arm. To determine the intervention's effectiveness, we evaluated the between-group proportion of patients with guideline-concordant care for process measures and the number of medication exposure days. We used a generalized linear mixed model (GLMM) with random practice intercepts to account for practice-level clustering. A logit link with a binomial family was used for process measures, while a log link with a Poisson family was used for medication prescription. Except for annual UACR testing guideline-concordance, we included time from baseline to last follow-up as a covariate in the binomial models and as an offset in the Poisson models to account for varying follow-up times across patients. The baseline time point was anchored at the first PCP visit after patient eligibility determination. All models were adjusted for age, sex, race, practice size, and baseline value of the guideline-concordant care outcome of interest as described in the study outcomes above. The UACR testing model was adjusted for whether the patient had undergone UACR testing in the 1 year before baseline. Medication exposure days at baseline were determined within 1 year before enrollment. This adjustment accounts for any preintervention differences in guideline adherence between the arms and helps ensure that the differences observed in the outcomes are more likely to be attributable to the intervention, rather than to baseline variations in care.

Predictive margins were calculated by group from the GLMM, and the relative benefit, defined as the ratio of the predictive margin in the intervention group to that in the usual care group, was estimated along with 95% CIs. Subgroup analyses were performed on age, sex, race, CKD stages, rural-urban location (rural-urban commuting area), and area deprivation index (ADI). The interaction between group and the subgroup variable was added to our GLMM analyses and tested for statistical significance to assess heterogeneity of intervention effects. To address missing data, we used the Multivariate Imputation by Chained Equations method.^29^ The imputation model included age, sex, race, baseline eGFR, BP, HbA1c, UACR, practice size, and medication exposure days. The results report pooled GLMM analyses after multiple imputation, ensuring that the analyses account for the uncertainty associated with missing data. *P* values < 0.05 were considered statistically significant. All analyses were performed using R version 4.3.1.^30^ Given multiplicity of outcomes, we applied the Benjamini–Hochberg method to control the false discovery rate (FDR) and provide adjusted *P* values for the nine different process measures analyzed. Both uncorrected and FDR-adjusted *P* values are presented.

For medication orders with missing order end date, we assessed the robustness of assumption of 1-year end date and performed sensitivity analyses by imputing alternative end dates, including (*1*) a 30-day assumption for the medication order end date; (*2*) a 90-day assumption for the medication order end date; and (*3*) a patient-specific median duration based on the patient's prior medication orders for same medication (for patients with no prior medication orders, we used the median duration across similar prescription orders with nonmissing end dates) (details in Supplemental Material).

## Results

### Population

K-CHAMP enrolled 1596 patients (754 intervention, 842 usual care) between May 2019 and November 2021 (enrollment details previously published).^15^ Overall mean age was 74 years, 928 (58%) were female, and 127 (8%) were Black (Table 2). Mean eGFR at baseline was 37±8 ml/min per 1.73 m^2^ and median UACR 85 (interquartile range, 15–422) mg/g. Characteristics of cohorts for each guideline-concordant care indicator are shown in Table 2. There were no substantial differences among patients enrolled from K-CHAMP practices versus usual care practices in each subcohort, and baseline medication exposure days were similar across arms (Supplemental Tables 1–6).

**Table 2:** Baseline characteristics of each cohort

Outcome Variable for the Cohort	BP, UACR	HbA1c	ACEi/ARB	Statin	SGLT2i	GLP-1RA
Variable	Overall, Mean±SD or *n* (%)	Diabetes (Type 1 and 2), Mean±SD or *n* (%)	UACR >300 mg/g; Diabetes and UACR 30–300 mg/g; Hypertension and UACR 30–300 mg/g, Mean±SD or *n* (%)	Age 40–75 yr, Mean±SD or *n* (%)	Type 2 Diabetes and eGFR ≥30 ml/min per 1.73 m^2^, Mean±SD or *n* (%)	Type 2 Diabetes, Mean±SD or *n* (%)
No. of patients in the cohort	*N*=1596	*N*=1027	*N*=932	*N*=789	*N*=834	*N*=1001
No. of PCP practices in the cohort	98	97	94	95	95	97
Age, yr	74±9	73±9	72±10	67±7	73±9	73±9
Female	928 (58)	558 (54)	463 (50)	430 (54)	430 (52)	549 (55)
**Race**						
Black	127 (8)	95 (9)	101 (11)	86 (11)	56 (7)	93 (9)
Others^a^	20 (1)	15 (2)	12 (1)	13 (2)	13 (2)	15 (2)
White	1449 (91)	917 (89)	819 (88)	690 (87)	765 (92)	893 (89)
**Ethnicity**						
Hispanic	9 (1)	9 (1)	9 (1)	4 (0.5)	7 (1)	9 (1)
Non-Hispanic	1579 (99)	1014 (99)	918 (99)	781 (99)	823 (99)	988 (99)
Number PCP visits in last 12 mo	4±2	4±2	4±3	4±2	4±2	4±2
BMI, kg/m^2^	32.2±7.4	33.3±7.4	32.5±7.4	33.8±8.1	33.5±7.4	33.3±7.4
Systolic BP, mm Hg	131±17	132±17	133±18	131±17	132±17	132±17
Diastolic BP, mm Hg	74±11	74±11	75±11	76±11	74±11	74±11
BP <130/80 mm Hg	684 (43)	415 (40)	346 (37)	316 (40)	336 (40)	402 (40)
BP <140/90 mm Hg	1164 (73)	725 (71)	626 (67)	560 (71)	591 (71)	709 (71)
Congestive heart failure	501 (31)	344 (33)	319 (34)	245 (31)	260 (31)	334 (33)
**Diabetes**						
Type 1	26 (2)	26 (3)	24 (3)	23 (2)	—	—
Type 2	1001 (63)	1001 (97)	692 (74)	541 (69)	834 (100)	1001 (100)
Hypertension	1512 (95)	1000 (97)	911 (98)	745 (94)	814 (98)	975 (97)
Cardiovascular disease	1248 (78)	866 (84)	767 (82)	616 (78)	705 (85)	845 (84)
CCI	6.8±2.9	8.1±2.4	7.3±2.8	7.1±3.0	8.0±2.3	8.0±2.3
ACEi/ARB	733 (46)	521 (51)	443 (48)	376 (48)	451 (54)	510 (51)
SGLT2i	42 (3)	40 (4)	30 (3)	29 (4)	38 (5)	40 (4)
GLP-1RA	80 (5)	80 (8)	59 (6)	60 (8)	71 (9)	80 (8)
SGLT2i and/or GLP-1RA	112 (7)	110 (11)	82 (9)	81 (10)	100 (12)	110 (11)
Mod- to high-intensity statin	808 (51)	571 (56)	488 (53)	423 (54)	471 (56)	555 (55)
No. of active meds	6±4	7±4	6±4	7±4	7±4	7±4
Creatinine, mg/dl	1.7±0.4	1.7±0.4	1.8±0.4	1.8±0.4	1.6±0.2	1.7±0.4
eGFR, ml/min per 1.73 m^2^	37±8	38±8	38±9	38±9	40±7	38±8
Serum potassium, mEq/L	4.4±0.5	4.5±0.5	4.4±0.5	4.4±0.5	4.5±0.4	4.5±0.5
HbA1c value, %	7.0±1.5	7.4±1.5	7.3±1.6	7.2±1.7	7.4±1.5	7.4±1.5
HbA1c <7%	727 (58)	478 (47)	395 (49.0)	351 (53)	376 (46)	471 (47)
UACR, mg/g, median (IQR)	85 (15–422)	121 (24–560)	286 (91–808)	117 (17–591)	116 (21–521)	117 (22–552)
Annual UACR	890 (56)	689 (67)	609 (65)	465 (59)	586 (70)	676 (68)
KFRE 5-yr risk %, median (IQR)	4.2 (2.2–9.3)	4.5 (2.4–9.9)	6.6 (3.6–13.9)	4.6 (2.2–11.5)	3.7 (2.1–7.0)	4.4 (2.4–9.6)
**CKD stage**						
2	10 (1)	7 (1)	7 (1)	8 (1)	7 (1)	7 (1)
3a	197 (12)	168 (16)	160 (17)	141 (18)	162 (19)	162 (16)
3b	1110 (70)	680 (66)	601 (64)	517 (66)	665 (80)	665 (66)
4	277 (17)	171 (17)	162 (17)	121 (15)	—	166 (17)
5	2 (0.1)	1 (0.1)	2 (0.2)	2 (0.3)	—	1 (0.1)
**Albuminuria stage**						
A1	509 (35)	280 (28)		237 (32)	238 (29)	278 (29)
A2	483 (33)	356 (36)	473 (51)	228 (31)	289 (35)	350 (36)
A3	459 (32)	360 (36)	459 (49)	271 (37)	288 (35)	342 (35)
ADI	67 (22)	68 (22)	68 (23)	69 (22)	68 (22)	68 (22)
**RUCA**						
Metropolitan	1225 (77)	782 (76)	744 (80)	609 (77)	630 (76)	761 (76)
Rural^b^	369 (23)	243 (24)	188 (20)	179 (23)	203 (24)	238 (24)

Missing baseline variables are summarized in Supplemental Table 7. ACEi, angiotensin-converting enzyme inhibitor; ADI, area deprivation index; ARB, angiotensin receptor blocker; BMI, body mass index; CCI, Charlson Comorbidity Index; GLP-1RA, glucagon-like peptide-1 receptor agonist; HbA1c, hemoglobin A1c; IQR, interquartile range; KFRE, kidney failure risk equation; PCP, primary care provider; RUCA, rural-urban commuting area; SGLT2i, sodium-glucose cotransporter-2 inhibitor; UACR, urine albumin-to-creatinine ratio.

a Other race includes American Indian, Chinese, Indian (Asian), other Asian, not specified, and those who declined to answer.

b Rural includes micropolitan and rural/town.

### Guideline-Concordant Care Process Measures

**Figure 1:**
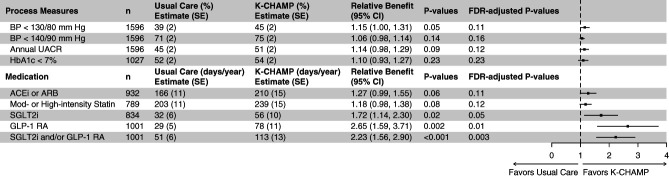
**Effect of K-CHAMP intervention on guideline-concordant process measures and medication exposure days as compared with usual care control.** Estimates and CIs were model-based; thus, raw numbers for numerator not presented. Relative benefit is the ratio of the predictive margin in the intervention group to that in the usual care group. ACEi, angiotensin-converting enzyme inhibitor; ARB, angiotensin receptor blocker; CI, confidence interval; FDR, false discovery rate; GLP-1RA, glucagon-like peptide-1 receptor agonists; HbA1c, hemoglobin A1c; K-CHAMP, Kidney Coordinated HeAlth Management Partnership; SGLT2i, sodium-glucose cotransporter-2 inhibitor; UACR, urine albumin-creatinine ratio.

After a median 17-month follow-up, there was no significant difference in achievement of BP <130/80 mm Hg (45% versus 39%; relative benefit 1.15; 95% CI, 1.00 to 1.31; FDR-adjusted *P* = 0.11), achievement of HbA1c <7% (54% versus 52%; relative benefit 1.10; 95% CI, 0.93 to 1.27; FDR-adjusted *P* = 0.23), or annual albuminuria testing (51% versus 45%; relative benefit 1.14; 95% CI, 0.98 to 1.29; FDR-adjusted *P* = 0.12) in intervention versus usual care (Figure 1). Exploratory analysis of BP goal of <140/90 was also not significantly different between arms. Among participants with diabetes, there was no difference in HbA1c monitoring—91% individuals in K-CHAMP versus 87% in usual care received at least yearly HbA1c monitoring (*P* = 0.27). There was no significant heterogeneity in intervention effect across subgroups of age, sex, race, CKD stage, ADI, and rural-urban commuting area (Supplemental Figures 1–4).

### Medication Exposure Days

Exposure days per year to ACEi/ARB and moderate-high intensity statin was not different between patients in the intervention arm compared with those in usual care (Figure 1). Patients in the intervention arm had significantly higher exposure days per year to SGLT2i (56 versus 32 days; relative benefit 1.72; 95% CI, 1.14 to 2.30; FDR-adjusted *P* = 0.05), GLP-1RA (78 versus 29 days; relative benefit 2.65; 95% CI, 1.59 to 3.71; FDR-adjusted *P* = 0.01), and SGLT2i and/or GLP-1RA (113 versus 51 days; relative benefit 2.23; 95% CI, 1.56 to 2.90; FDR-adjusted *P* = 0.003) compared with usual care. Subgroup analyses showed that K-CHAMP intervention significantly increased SGLT2i exposure days among women and in patients of White race (Supplemental Figure 7). Similarly, the intervention significantly increased SGLT2i and/or GLP-1RA exposure days among patients of White race (Supplemental Figure 9). There was no significant heterogeneity in intervention effect on exposure days to ACEi/ARB patients with albuminuria, moderate-high intensity statin, or GLP-1RA individually, across subgroups of age, sex, race, CKD stage, ADI, and rural/urban-living patients (Supplemental Figures 5, 6, and 8). Proportion of medication orders with missing end dates ranged from 8% to 14% and was similar between the intervention and control arms (Supplemental Table 8). Specifically, for ACEi/ARBs and statins, which had the highest number of missing medication order end date, the median (first quartile, third quartile) number of exposure days among those without missing medication order end date was 365 (286–365) days and 365 (334–365) days, respectively, thus supporting our assumption of 1 year. In sensitivity analyses, using medication order end date of 30 days, 90 days, or patient-specific median historical medication order duration yielded similar conclusions (Supplemental Figures 10–12).

### Temporal Trend in Guideline-Concordant Medication Prescription

**Figure 2:**
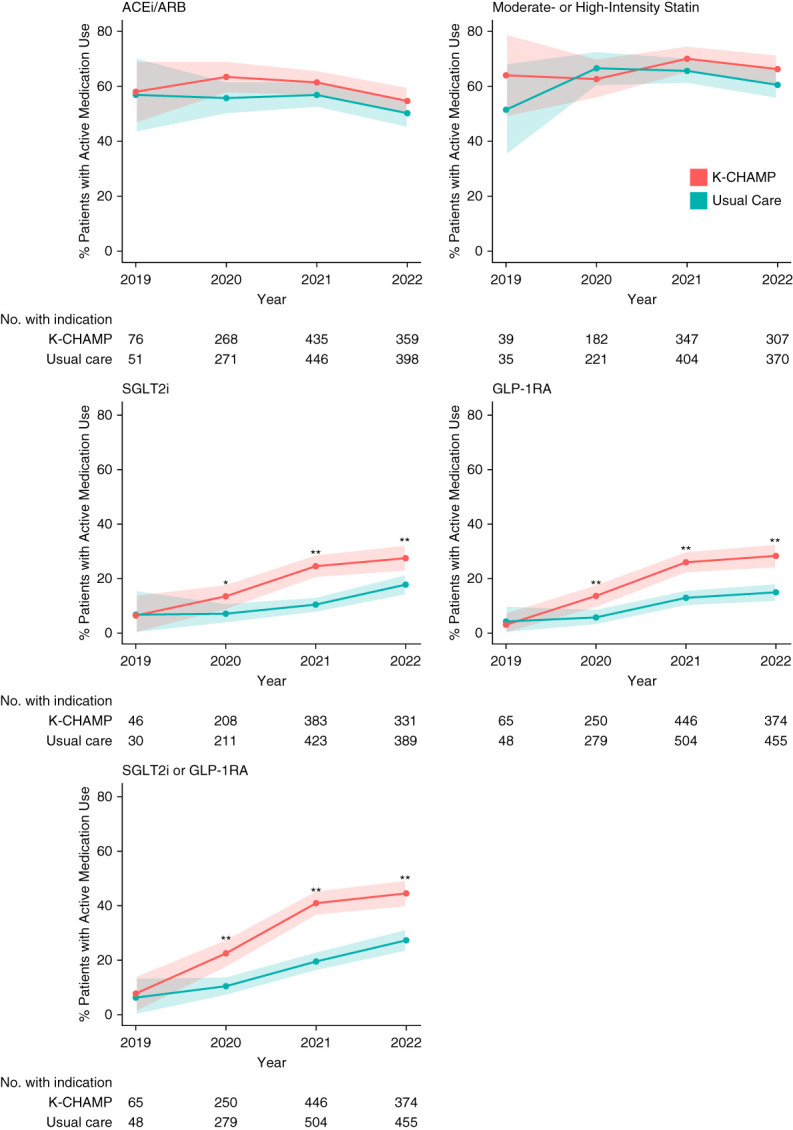
**Temporal trends in use of guideline-concordant medications in K-CHAMP versus usual care arm.** *P* value represents difference in proportion of patients in each arm during the same time interval. *P < 0.05, **P < 0.01. Shaded area represents pointwise 95% confidence bands. No. with indication is number of patients in evaluated cohort per year, as designated in Table 1.

Proportion of patients with an active medication prescription for guideline-concordant medications during the study period are displayed in Figure 2 and Supplemental Table 9. At study initiation in 2019, proportion of patients prescribed SGLT2i and/or GLP-1RA was similar between arms (8% [*N*=5/65] versus 6% [*N*=3/48], respectively; rate ratio 1.23; 95% CI, 0 to 2.99; *P* = 0.8). By 2022, significantly higher proportion of intervention arm patients were prescribed SGLT2i and/or GLP-1RA compared with usual care (44% [*N*=166/374] versus 27% [*N*=124/455], respectively; rate ratio 1.63; 95% CI, 1.32 to 1.94; *P* < 0.001). Similarly, a significantly higher proportion of patients were prescribed SGLT2i or GLP-1RA individually. Prescription of ACEi/ARB in patients with albuminuria or moderate-high intensity statin did not differ between arms.

## Discussion

Our secondary analysis of the K-CHAMP trial demonstrates the effectiveness of a population health management approach to accelerate implementation of new evidence-based treatments for CKD in the primary care setting. Patients in the intervention arm were almost twice as likely to be prescribed SGLT2i and/or GLP-1RA than those in the usual care arm over a median 17-month follow-up period. Although there were positive trends in favor of K-CHAMP intervention for improved BP control, glycemic control, annual albuminuria testing, and exposure days to ACEi/ARB in patients with albuminuria and moderate-high intensity statin, these did not reach statistical significance.

With the advent of transformative clinical management guidelines for CKD, there is an unprecedented urgency to enhance implementation in clinical practice. Unfortunately, early implementation data from several health systems across the United States suggests only a modest increase in their clinical uptake. For instance, data from 2019 to 2020 from the Veteran Affairs Health System showed that the use of SGLT2i was 11%–12% and GLP-1RA was 8%–10% in patients with type 2 diabetes and CKD or atherosclerotic cardiovascular disease.^31–34^ During the same time period, SGLT2i use was 7% and GLP-1RA use was 4%–6% in patients with type 2 diabetes and CKD in the usual care arm in our trial. These low rates are not surprising given insurance preauthorization requirements and higher drug costs in the non–Veteran Affairs setting and are comparable with those reported during the same period among patients from other community practices.^35^ Expected secular trends revealed increased prescription of these medications in our usual care arm, reflecting increased PCP awareness, accessibility, and affordability. These rising utilization trends were also seen in other health care systems across the United States—13%–14% for SGLT2i or GLP-1RA alone and 23% for either agent in March 2021.^35^ By contrast, SGLT2i and GLP-1RA use in the K-CHAMP intervention arm was nearly twice as high. Among patients with type 2 diabetes and CKD, 28% were prescribed SGLT2i or GLP-1RA individually, and 44% were prescribed SGLT2i and/or GLP-1RA by July 2022. In addition, patients receiving the K-CHAMP intervention had almost 2–3 times higher rate of exposure days to SGLT2i and GLP-1RA as compared with usual care. Our intervention's novel population health, multidisciplinary team approach strategically overcame barriers at multiple levels to facilitate clinical practice change.^36,37^ We believe our intervention mitigated PCPs' therapeutic inertia, augmented prescribing confidence and knowledge through ongoing nephrologist and pharmacist support, and academic detailing. We educated PCPs on expected changes, such as an early transient reduction in eGFR after SGLT2i initiation, likely decreasing the chances of medication discontinuation, which is reported to be as high as 65% in this population.^33^ Patient education about CKD and its complications, treatment benefits, and best practices to minimize side effects likely facilitated patient acceptance of new medications. In addition, our pharmacists addressed payor-level and health system–level barriers by providing resources for medication assistance programs. This multidisciplinary team was a critical extension to PCP offices, which are often inundated with a milieu of patient care tasks but too little staff or time. These unique aspects of our intervention helped us change clinical care in a relatively short median follow-up of 17 months and shorten the often-quoted implementation gap of 17 years from research to clinical practice.^38^

Several recent implementation efforts to increase clinical adoption of newer guidelines have been studied in various settings. Thomas *et al.* described outcomes from their Cardiometabolic Center of Excellence, which used a collaborative care, multispecialty team–based approach with nurse navigators, pharmacists, and educators. Among the 129 patients with type 2 diabetes and cardiovascular disease seen in this clinic, there was a significantly higher utilization rate of SGLT2i/GLP-1RA as compared with a propensity-matched control group.^39^ The recent Coordinating Cardiology Clinics Randomized Trial of Interventions to Improve Outcomes–Diabetes used a similar collaborative care, multidisciplinary implementation approach in cardiology clinics and showed almost three times higher rates of SGLT2i/GLP-1RA in patients with type 2 diabetes and atherosclerotic cardiovascular disease as compared with controls.^40^ Both these studies included patients seen in specialty clinics, which may have the advantage of having providers who are more familiar with new guidelines and patients who are more engaged. Our study extends these findings to a primary care setting and provides evidence on a novel EHR-based, multidisciplinary, population health implementation strategy for newer therapeutics in the CKD population.

K-CHAMP intervention showed trends toward improvement in other guideline-concordant process measures, such as BP and glycemic control, and other renoprotective medication use, but narrowly missed achieving statistical significance. We suspect that the increased emphasis on newer therapeutics and changing guidelines may have shifted PCPs' focus away from these other guideline-concordant aspects of CKD care. Perhaps even more importantly, coronavirus disease 2019 pandemic–related challenges in primary care, which shifted focus from chronic disease management to acute illness, vaccination uptake, and social needs, likely affected the outcomes of our real-world implementation study. Furthermore, despite greater exposure days to SGLT2i and/or GLP-1RA in the intervention arm, the overall exposure was only 113 days and likely not long enough to observe treatment effect on kidney outcomes, given that the median time for treatment effect was 2–3.5 years in recent trials of these medications.^41,42^ All these factors may have contributed to lack of effect of K-CHAMP intervention on CKD progression/kidney failure in our trial.

Our study was strengthened by including patients from 13 counties and 24% patients from rural areas, thus facilitating equitable access to a large region, with minimal patient burden. We anticipate a scalable and equitable strategy, such as K-CHAMP, may help achieve Advancing American Kidney Health initiative's goal of reducing incidence of kidney failure by 25% by 2030.^43^ Future studies are needed to confirm the long-term effect of such a population health management approach in an era of more unified guidelines, affordable drug costs, and increased availability of safety data of these newer medications in older adults.

Despite these strengths, our study has notable limitations. Our cohort had rural-urban diversity, but the communities in Western Pennsylvania lacked racial and ethnic diversity, potentially limiting the generalizability of our findings. Although our results suggest racial disparity and significantly higher exposure days to SGLT2i and/or GLP-1RA with the intervention among patients of White race, our results should be interpreted with caution because <10% of our population was of non-White race. Large CKD cohort studies indicate that racial and ethnic minority groups have up to 15%–20% lower rate of prescription of SGLT2i and GLP-1RA.^31,44^ Future studies are needed to evaluate the impact of this program in addressing these disparities in a racially and ethnically diverse population. In addition, we analyzed only medication prescription data in EHR, which does not link to prescription fill data; thus, medication exposure days may not accurately reflect patient medication adherence. Moreover, our results do not account for medication side effects, such as statin-induced myopathy, or hesitancies for initiating ACEi/ARB such as hypotension, hyperkalemia, or eGFR <30 ml/min per 1.73 m^2^. EHR medication prescription data were also limited by missing medication order end dates in approximately 8%–14% of orders; assumption of 1-year prescription duration for those with missing end dates may underestimate variability in the data. This could have affected our ability to detect significant differences between the intervention and control groups. However, based on the results of our sensitivity analyses, we believe that this potential source of bias did not substantially affect our primary conclusions. Our analysis included recommendations from the Kidney Disease Improving Global Outcomes 2020 Clinical Practice Guideline for Diabetes Management in Chronic Kidney Disease, which was the most updated version during the study period. However, this guideline was updated in 2022 to include lower eGFR threshold for SGLT2i and addition of finerenone in diabetic kidney disease.^41,45,46^ In addition, for laboratory tests such as UACR and HbA1c, we only have EHR data for completed tests with results. Thus, we cannot discern whether the laboratory test was ordered, but not completed by the patient, or never ordered. Moreover, if a laboratory test was never ordered at a PCP visit, we cannot determine whether this was because it was an acute/sick visit (*e.g*., hospital follow-up, upper respiratory infection), as opposed to a planned, well-visit. In addition, our internal prediction model to identify high-risk CKD has not been externally validated. Finally, the multifaceted nature of intervention limits our ability to examine which component was most effective. However, as recommended by Kidney Disease Improving Global Outcomes, multidisciplinary team care is often needed to care for complex patients with CKD.^47^

Nephrology is at the cusp of exciting transformative clinical practice change for patients with CKD.^48^ A population health management–based multidisciplinary team approach can be a successful implementation strategy to accelerate uptake of emerging evidence-based treatments across a large health system.

## Supplementary Material

## Data Availability

Anonymized data created for the study are or will be available in a persistent repository upon publication. Partial restrictions to the data and/or materials apply. Clinical Trial Data. GitHub. https://github.com/Renal-Electrolyte/KChamp_data_sharing.git. Data will be made available to investigators with a methodologically sound proposal, with institutional review board approval and demonstration of resources to be able to undertake the proposed analyses, for a wide range of purposes subject to review and approval by the study's executive committee, and after approval of the proposal, consistent with guidelines of the University of Pittsburgh. Contact Manisha Jhamb at jhambm@upmc.edu.

## Supplemental Material

Supplemental Table 1. Baseline characteristics of patients enrolled from control versus K-CHAMP practices for BP, UACR cohort (*n*=1596).

Supplemental Table 2. Baseline characteristics of patients enrolled from control versus K-CHAMP practices for HbA1c cohort (*n*=1027).

Supplemental Table 3. Baseline characteristics of patients enrolled from control versus K-CHAMP practices for ACEi/ARB cohort (*n*=932).

Supplemental Table 4. Baseline characteristics of patients enrolled from control versus K-CHAMP practices for statin cohort (*n*=789).

Supplemental Table 5. Baseline characteristics of patients enrolled from control versus K-CHAMP practices for GLP-1RA cohort (*n*=1001).

Supplemental Table 6. Baseline characteristics of patients enrolled from control versus K-CHAMP practices for SGLT2i cohort (*n*=834).

Supplemental Table 7. Number of participants with missing baseline variables.

Supplemental Table 8. Number of medication orders with missing dates by arm.

Supplemental Table 9. Proportion of patients prescribed guideline-concordant medications in intervention versus usual care arm by year.

Supplemental Figure 1. Effect of K-CHAMP intervention on BP control <130/80 mm Hg compared with usual care control by subgroup.

Supplemental Figure 2. Effect of K-CHAMP intervention on BP control <140/90 mm Hg compared with usual care control by subgroup.

Supplemental Figure 3. Effect of K-CHAMP intervention on annual albuminuria testing compared with usual care control by subgroup.

Supplemental Figure 4. Effect of K-CHAMP intervention on glycemic control compared with usual care control by subgroup.

Supplemental Figure 5. Effect of K-CHAMP intervention on ACEi/ARB exposure days compared with usual care control by subgroup.

Supplemental Figure 6. Effect of K-CHAMP intervention on moderate-high intensity statin exposure days compared with usual care control by subgroup.

Supplemental Figure 7. Effect of K-CHAMP intervention on SGLT2i exposure days compared with usual care control by subgroup.

Supplemental Figure 8. Effect of K-CHAMP intervention on GLP-1RA exposure days compared with usual care control by subgroup.

Supplemental Figure 9. Effect of K-CHAMP intervention on SGLT2i and/or GLP-1RA exposure days compared with usual care control by subgroup.

Supplemental Figure 10. Sensitivity analyses results: 30 days imputation for prescription orders with missing end dates.

Supplemental Figure 11. Sensitivity analyses results: 90 days imputation for prescription orders with missing end dates.

Supplemental Figure 12. Sensitivity analyses results: patient-specific median duration imputation for prescription orders with missing end dates.
