# Supplementary material for: Population Health Management and Guideline-Concordant Care in CKD: A Secondary Analysis of Kidney Coordinated HeAlth Management Partnership
Source: J Am Soc Nephrol. 2024 Nov 1;36(5):869–81. doi: 10.1681/ASN.0000000544 (PMC12059108; doi:10.1681/ASN.0000000544)
Supplement: Supplementary file 1 [file jasn-36-869-s001.pdf]

ASN American Society of Nephrology | JASN Journal of the American Society of Nephrology | KIDNEY360 Accessing Our World From Every Angle | CJASN Clinical Journal of the American Society of Nephrology

# ASN Journal Disclosure Form

As per ASN journal policy, I have disclosed any financial relationships or commitments I have held in the past 36 months as included below. I have listed my Current Employer below to indicate there is a relationship requiring disclosure. If no relationship exists, my Current Employer is not listed.

A. Alghwiri reports the following:
Employer: University of Pittsburgh

I understand that the information above will be published within the journal article, if accepted, and that failure to comply and/or to accurately and completely report the potential financial conflicts of interest could lead to the following: 1) Prior to publication, article rejection, or 2) Post-publication, sanctions ranging from, but not limited to, issuing a correction, reporting the inaccurate information to the authors' institution, banning authors from submitting work to ASN journals for varying lengths of time, and/or retraction of the published work.

Name: Alaa A. Alghwiri
Manuscript ID: JASN-2024-000452R1
Manuscript Title: Effect of population health management on guideline concordant care in CKD: Secondary analysis of K-CHAMP  cluster randomized trial
Date of Completion: July 21, 2024
Disclosure Updated Date: May 16, 2024

ASN American Society of Nephrology | JASN Journal of the American Society of Nephrology | KIDNEY360 Accessing Our World From Every Angle | CJASN Clinical Journal of the American Society of Nephrology

# ASN Journal Disclosure Form

As per ASN journal policy, I have disclosed any financial relationships or commitments I have held in the past 36 months as included below. I have listed my Current Employer below to indicate there is a relationship requiring disclosure. If no relationship exists, my Current Employer is not listed.

G. Fischer reports the following:
Employer: University of Pittsburgh and University of Pittsburgh Physicians; Consultancy: Me -- DJS Associates, Inc --contacted through them expert witness for State Medical Board; Wife - Integrated Care Corp -- through them provides home based OT services for early intervention as contractor; and Ownership Interest: ATT, Lumen, Nokia, Verizon, Warner Bros, Comcast, Vodafone.

I understand that the information above will be published within the journal article, if accepted, and that failure to comply and/or to accurately and completely report the potential financial conflicts of interest could lead to the following: 1) Prior to publication, article rejection, or 2) Post-publication, sanctions ranging from, but not limited to, issuing a correction, reporting the inaccurate information to the authors' institution, banning authors from submitting work to ASN journals for varying lengths of time, and/or retraction of the published work.

Name: Gary Fischer
Manuscript ID: JASN-2024-000452R1
Manuscript Title: "Effect of population health management on guideline concordant care in CKD: Secondary analysis of K-CHAMP cluster randomized trial
Date of Completion: July 30, 2024
Disclosure Updated Date: July 30, 2024

ASN American Society of Nephrology | JASN Journal of the American Society of Nephrology | KIDNEY360 Accessing Our World From Every Angle | CJASN Clinical Journal of the American Society of Nephrology

# ASN Journal Disclosure Form

As per ASN journal policy, I have disclosed any financial relationships or commitments I have held in the past 36 months as included below. I have listed my Current Employer below to indicate there is a relationship requiring disclosure. If no relationship exists, my Current Employer is not listed.

Z. Han reports the following:
Employer: University of Pittsburgh

I understand that the information above will be published within the journal article, if accepted, and that failure to comply and/or to accurately and completely report the potential financial conflicts of interest could lead to the following: 1) Prior to publication, article rejection, or 2) Post-publication, sanctions ranging from, but not limited to, issuing a correction, reporting the inaccurate information to the authors' institution, banning authors from submitting work to ASN journals for varying lengths of time, and/or retraction of the published work.

Name: Zhuoheng Han
Manuscript ID: JASN-2024-000452R3
Manuscript Title: Effect of Population Health Management on Guideline Concordant Care in CKD: Secondary Analysis of K-CHAMP Cluster Randomized Trial
Date of Completion: October 17, 2024
Disclosure Updated Date: May 21, 2024

ASN
American Society of Nephrology
JASN
Journal of the American Society of Nephrology
KIDNEY360
Accessing Our World From Every Angle
CJASN
Clinical Journal of the American Society of Nephrology

# ASN Journal Disclosure Form

As per ASN journal policy, I have disclosed any financial relationships or commitments I have held in the past 36 months as included below. I have listed my Current Employer below to indicate there is a relationship requiring disclosure. If no relationship exists, my Current Employer is not listed.

M. Jhamb reports the following:
Employer: University of Pittsburgh and University of Pittsburgh Medical Center; Consultancy: Xcenda, LLC; Boehringer Ingelheim LLC, CKD Leaders Networks, Eli-Lilly; Research Funding: NIH, Dialysis Clinic, Inc., Bayer LLC, Pfizer, CKD Leaders Network; and Other Interests or Relationships: Member of ASN and National Kidney Foundation.

I understand that the information above will be published within the journal article, if accepted, and that failure to comply and/or to accurately and completely report the potential financial conflicts of interest could lead to the following: 1) Prior to publication, article rejection, or 2) Post-publication, sanctions ranging from, but not limited to, issuing a correction, reporting the inaccurate information to the authors' institution, banning authors from submitting work to ASN journals for varying lengths of time, and/or retraction of the published work.

Name: Manisha Jhamb
Manuscript ID: JASN-2024-000452R1
Manuscript Title: Effect of population health management on guideline concordant care in CKD: Secondary analysis of K-CHAMP cluster randomized trial
Date of Completion: July 20, 2024
Disclosure Updated Date: April 29, 2024

# ASN Journal Disclosure Form

As per ASN journal policy, I have disclosed any financial relationships or commitments I have held in the past 36 months as included below. I have listed my Current Employer below to indicate there is a relationship requiring disclosure. If no relationship exists, my Current Employer is not listed.

L. Lavenburg reports the following:
Research Funding: American Heart Association; Pfizer (investigator initiated study)

I understand that the information above will be published within the journal article, if accepted, and that failure to comply and/or to accurately and completely report the potential financial conflicts of interest could lead to the following: 1) Prior to publication, article rejection, or 2) Post-publication, sanctions ranging from, but not limited to, issuing a correction, reporting the inaccurate information to the authors' institution, banning authors from submitting work to ASN journals for varying lengths of time, and/or retraction of the published work.

Name: Linda-Marie Ustaris Lavenburg
Manuscript ID: JASN-2024-000452R3
Manuscript Title: Effect of Population Health Management on Guideline Concordant Care in CKD: Secondary Analysis of K-CHAMP Cluster Randomized Trial
Date of Completion: October 17, 2024
Disclosure Updated Date: August 30, 2024

ASN American Society of Nephrology | JASN Journal of the American Society of Nephrology | KIDNEY360 Accessing Our World From Every Angle | CJASN Clinical Journal of the American Society of Nephrology

# ASN Journal Disclosure Form

As per ASN journal policy, I have disclosed any financial relationships or commitments I have held in the past 36 months as included below. I have listed my Current Employer below to indicate there is a relationship requiring disclosure. If no relationship exists, my Current Employer is not listed.

M. Mosslemi has nothing to disclose.

I understand that the information above will be published within the journal article, if accepted, and that failure to comply and/or to accurately and completely report the potential financial conflicts of interest could lead to the following: 1) Prior to publication, article rejection, or 2) Post-publication, sanctions ranging from, but not limited to, issuing a correction, reporting the inaccurate information to the authors' institution, banning authors from submitting work to ASN journals for varying lengths of time, and/or retraction of the published work.

Name: Mitra Mosslemi
Manuscript ID: JASN-2024-000452R1
Manuscript Title: Effect of population health management on guideline concordant care in CKD: Secondary analysis of K-CHAMP cluster randomized trial
Date of Completion: July 22, 2024
Disclosure Updated Date: May 20, 2024

ASN American Society of Nephrology
JASN Journal of the American Society of Nephrology
KIDNEY360 Accessing Our World From Every Angle
CJASN Clinical Journal of the American Society of Nephrology

# ASN Journal Disclosure Form

As per ASN journal policy, I have disclosed any financial relationships or commitments I have held in the past 36 months as included below. I have listed my Current Employer below to indicate there is a relationship requiring disclosure. If no relationship exists, my Current Employer is not listed.

T. Nolin reports the following:
Employer: University of Pittsburgh; Consultancy: MediBeacon; CytoSorbents; Mineralys; Ownership Interest: Healthmap Solutions; Patents or Royalties: McGraw-Hill Education; and Advisory or Leadership Role: Healthmap Solutions-Scientific Advisory Board; American College of Clinical Pharmacology-Treasurer; McGraw-Hill-Editor.

I understand that the information above will be published within the journal article, if accepted, and that failure to comply and/or to accurately and completely report the potential financial conflicts of interest could lead to the following: 1) Prior to publication, article rejection, or 2) Post-publication, sanctions ranging from, but not limited to, issuing a correction, reporting the inaccurate information to the authors' institution, banning authors from submitting work to ASN journals for varying lengths of time, and/or retraction of the published work.

Name: Thomas D. Nolin
Manuscript ID: JASN-2024-000452R3
Manuscript Title: Effect of Population Health Management on Guideline Concordant Care in CKD: Secondary Analysis of K-CHAMP Cluster Randomized Trial
Date of Completion: October 19, 2024
Disclosure Updated Date: October 19, 2024

ASN American Society of Nephrology | JASN Journal of the American Society of Nephrology | KIDNEY360 Accessing Our World From Every Angle | CJASN Clinical Journal of the American Society of Nephrology

# ASN Journal Disclosure Form

As per ASN journal policy, I have disclosed any financial relationships or commitments I have held in the past 36 months as included below. I have listed my Current Employer below to indicate there is a relationship requiring disclosure. If no relationship exists, my Current Employer is not listed.

B. Rollman reports the following:
Employer: University of Pittsburgh School of Medicine

I understand that the information above will be published within the journal article, if accepted, and that failure to comply and/or to accurately and completely report the potential financial conflicts of interest could lead to the following: 1) Prior to publication, article rejection, or 2) Post-publication, sanctions ranging from, but not limited to, issuing a correction, reporting the inaccurate information to the authors' institution, banning authors from submitting work to ASN journals for varying lengths of time, and/or retraction of the published work.

Name: Bruce L. Rollman
Manuscript ID: JASN-2024-000452R1
Manuscript Title: "Effect of population health management on guideline concordant care in CKD: Secondary analysis of K-CHAMP cluster randomized trial,"
Date of Completion: July 30, 2024
Disclosure Updated Date: March 4, 2024

ASN American Society of Nephrology | JASN Journal of the American Society of Nephrology | KIDNEY360 Accessing Our World From Every Angle | CJASN Clinical Journal of the American Society of Nephrology

# ASN Journal Disclosure Form

As per ASN journal policy, I have disclosed any financial relationships or commitments I have held in the past 36 months as included below. I have listed my Current Employer below to indicate there is a relationship requiring disclosure. If no relationship exists, my Current Employer is not listed.

M. Weltman reports the following:
Employer: University of Pittsburgh School of Pharmacy

I understand that the information above will be published within the journal article, if accepted, and that failure to comply and/or to accurately and completely report the potential financial conflicts of interest could lead to the following: 1) Prior to publication, article rejection, or 2) Post-publication, sanctions ranging from, but not limited to, issuing a correction, reporting the inaccurate information to the authors' institution, banning authors from submitting work to ASN journals for varying lengths of time, and/or retraction of the published work.

Name: Melanie R. Weltman
Manuscript ID: JASN-2024-000452R1
Manuscript Title: Effect of population health management on guideline concordant care in CKD: Secondary analysis of K-CHAMP cluster randomized trial
Date of Completion: July 22, 2024
Disclosure Updated Date: May 14, 2024

ASN American Society of Nephrology | JASN Journal of the American Society of Nephrology | KIDNEY360 Accessing Our World From Every Angle | CJASN Clinical Journal of the American Society of Nephrology

# ASN Journal Disclosure Form

As per ASN journal policy, I have disclosed any financial relationships or commitments I have held in the past 36 months as included below. I have listed my Current Employer below to indicate there is a relationship requiring disclosure. If no relationship exists, my Current Employer is not listed.

J. Yabes reports the following:
Employer: University of Pittsburgh

I understand that the information above will be published within the journal article, if accepted, and that failure to comply and/or to accurately and completely report the potential financial conflicts of interest could lead to the following: 1) Prior to publication, article rejection, or 2) Post-publication, sanctions ranging from, but not limited to, issuing a correction, reporting the inaccurate information to the authors' institution, banning authors from submitting work to ASN journals for varying lengths of time, and/or retraction of the published work.

Name: Jonathan Guerrero Yabes
Manuscript ID: JASN-2024-000452R3
Manuscript Title: Effect of Population Health Management on Guideline Concordant Care in CKD: Secondary Analysis of K-CHAMP Cluster Randomized Trial
Date of Completion: October 17, 2024
Disclosure Updated Date: May 8, 2024
