# Supplementary material for: Population Health Management and Guideline-Concordant Care in CKD: A Secondary Analysis of Kidney Coordinated HeAlth Management Partnership
Source: J Am Soc Nephrol. 2024 Nov 1;36(5):869–81. doi: 10.1681/ASN.0000000544 (PMC12059108; doi:10.1681/ASN.0000000544)
Supplement: Supplementary file 2 [file jasn-36-869-s002.pdf]

Supplemental Material

Population Health Management and Guideline-Concordant Care in CKD: A Secondary Analysis of K-CHAMP

Melanie R. Weltman*, PharmD[1,2], Linda-Marie U. Lavenburg*, DO, MS[1], Zhuoheng Han, MS[1], Alaa A. Alghwiri, PhD[1], Mitra Mosslemi, MS[6], Bruce L. Rollman, MD, MPH[3,4], Gary S Fischer, MD[3], Thomas D. Nolin, PharmD, PhD[1,2], Jonathan G. Yabes, PhD[5], Manisha Jhamb, MD, MPH[1]

Supplemental Table 1. Baseline characteristics of patients enrolled from control versus K-CHAMP practices for BP, UACR cohort (*n*=1596)

Supplemental Table 2. Baseline characteristics of patients enrolled from control versus K-CHAMP practices for HbA1c cohort (*n*=1027)

Supplemental Table 3. Baseline characteristics of patients enrolled from control versus K-CHAMP practices for ACEi/ARB cohort (*n*=932)

Supplemental Table 4. Baseline characteristics of patients enrolled from control versus K-CHAMP practices for statin cohort (*n*=789)

Supplemental Table 5. Baseline characteristics of patients enrolled from control versus K-CHAMP practices for GLP-1 RA cohort (*n*=1001)

Supplemental Table 6. Baseline characteristics of patients enrolled from control versus K-CHAMP practices for SGLT2i cohort (*n*=834)

Supplemental Table 7. Number of participants with missing baseline variables

Supplemental Table 8. Number of Medication Orders with Missing Dates by Arm

Supplemental Table 9. Proportion of patients prescribed guideline-concordant medications in intervention versus usual care arm by year

Supplemental Figure 1. Effect of K-CHAMP intervention on BP control < 130/80 mmHg compared to usual care control by subgroup

Supplemental Figure 2. Effect of K-CHAMP intervention on BP control < 140/90 mmHg compared to usual care control by subgroup

Supplemental Figure 3. Effect of K-CHAMP intervention on annual albuminuria testing compared to usual care control by subgroup

Supplemental Figure 4. Effect of K-CHAMP intervention on glycemic control compared to usual care control by subgroup

Supplemental Figure 5. Effect of K-CHAMP intervention on ACEi/ARB exposure days compared to usual care control by subgroup

Supplemental Figure 6. Effect of K-CHAMP intervention on moderate-high intensity statin exposure days compared to usual care control by subgroup

Supplemental Figure 7. Effect of K-CHAMP intervention on SGLT2i exposure days compared to usual care control by subgroup

Supplemental Figure 8. Effect of K-CHAMP intervention on GLP-1RA exposure days compared to usual care control by subgroup

Supplemental Figure 9. Effect of K-CHAMP intervention on SGLT2i and/or GLP-1RA exposure days compared to usual care control by subgroup

Supplemental Figure 10. Sensitivity analyses results: 30 days imputation for prescription orders with missing end dates

Supplemental Figure 11. Sensitivity analyses results: 90 days imputation for prescription orders with missing end dates

Supplemental Figure 12. Sensitivity analyses results: Patient-specific median duration imputation for prescription orders with missing end dates

Supplemental Table 1. Baseline characteristics of patients enrolled from control versus K-CHAMP practices for BP, UACR cohort (*n*=1596)

Variable	Control (*N* = 842)<br><br>Mean ± SD or *n* (%)	K-Champ (*N* = 754)<br><br>Mean ± SD or *n* (%)	Standardized Bias
**Age, years**	73 ± 9	74 ± 9	4.17
**Female**	519 (6)	409 (54)	15.02
**Race**			
Black	76 (9)	51 (7)	8.40
White	753 (89)	696 (92)	10.00
Others*	13 (2)	7 (1)	5.57
**Ethnicity**			
Hispanic	5 (1)	4 (1)	0.88
Non-Hispanic	831 (99)	748 (99)	0.88
**Number PCP visits in last 12 months**	4 ± 2	4 ± 3	10.99
**BMI, kg/m^2**	32.1 ± 7.3	32.3 ± 7.6	2.61
**Systolic BP, mmHg**	132 ± 17	131 ± 17	2.99
**Diastolic BP, mmHg**	74 ± 11	74 ± 10	4.90
**BP < 130/80 mmHg**	359 (43)	325 (43)	0.94
**BP < 140/90 mmHg**	605 (72)	559 (74)	5.15
**CHF**	249 (30)	252 (33)	8.29
**DM**			
Type 1	12 (1)	14 (2)	3.40
Type 2	534 (63)	467 (62)	3.07
**HTN**	801 (95)	711 (94)	3.73
**CVD**	653 (78)	595 (79)	3.29
**CCI**	6.8 ± 3.0	6.8 ± 2.8	2.66
**ACEi/ARB**	391 (46)	342 (45%)	2.17
**SGLT2i**	18 (2)	24 (3)	6.50
**GLP-1 RA**	32 (4)	48 (6)	11.70
**SGLT2i and/or GLP-1 RA**	45 (5)	67 (9)	13.81
**Mod- to High-intensity statin**	427 (51)	381 (51)	0.36
**Number of active meds**	6 ± 4	6 ± 3	5.13
**Cr, mg/dL**	1.7 ± 0.4	1.7 ± 0.4	0.33
**eGFR, mL/min/1.73m^2**	37 ± 8	37 ± 8	6.69
**Serum potassium level, meq/L**	4.4 ± 0.5	4.4 ± 0.4	1.27
**HbA1c Value, %**	7.0 ± 1.5	7.0 ± 1.5	0.13
**HbA1c < 7%**	400 (59)	327 (56)	7.01
**UACR, mg/g Median (IQR)**	85 (14, 410)	86 (16, 434)	1.17
**Annual UACR**	450 (53)	440 (58)	9.90
**KFRE-5 year risk % Median (IQR)**	4.1 (2.0, 9.3)	4.2 (2.3, 9.1)	5.33
**CKD stages**			
2	4 (0.5)	6 (1)	4.04
3a	99 (12)	98 (13)	3.76
3b	590 (70)	520 (69)	2.40
4	147 (17)	130 (17)	0.57
5	2 (0.2)	0 (0)	6.90
**Albuminuria stage**			

A1	276 (37)	233 (33)	6.66
A2	245 (32)	238 (34)	3.51
A3	233 (31)	226 (32)	3.27
**ADI**	68 ± 22	66 ± 22	7.73
**RUCA**			
Metropolitan	658 (78)	567 (75)	6.97
Rural**	183 (22)	186 (25)	6.97

*Other race includes American Indian, Chinese, Indian (Asian), Other Asian, Not specified, and those who declined to answer

**Rural includes micropolitan and rural/town

Abbreviations: BP, blood pressure; UACR, urine albumin-to-creatinine ratio; HbA1c, hemoglobin A1c; ACEi, angiotensin-converting enzyme inhibitor; ARB, angiotensin II receptor blocker; GLP-1RA, glucagon-like peptide-1 receptor agonist; SGLT2i, sodium–glucose cotransporter-2 inhibitor; SD, standard deviation; eGFR, estimated glomerular filtration rate; PCP, primary care provider; BMI, body mass index; CCI, Charlson comorbidity index; IQR, interquartile range; KFRE, kidney failure risk equation; ADI, area deprivation index; RUCA, rural-urban commuting area

Supplemental Table 2. Baseline characteristics of patients enrolled from control versus K-CHAMP practices for HbA1c cohort (*n*=1027)

**Variable**	**Control (*N* = 546)**<br><br>Mean ± SD or *n* (%)	**K-Champ (*N* = 481)**<br><br>Mean ± SD or *n* (%)	**Standardized Bias**
**Age, years**	72 ± 9	73 ± 9	4.04
**Female**	316 (58)	242 (50)	15.22
**Race**			
Black	54 (10)	41 (9)	4.73
White	483 (88)	434 (90)	5.73
Others*	0 (2)	6 (1)	3.36
**Ethnicity**			
Hispanic	5 (1)	4 (1)	0.94
Non-Hispanic	538 (99)	476 (99)	0.94
**Number PCP visits in last 12 months**	4 ± 2	4 ± 3	9.90
**BMI, kg/m^2**	33.1 ± 7.3	33.5 ± 7.5	6.35
**Systolic BP, mmHg**	133 ± 18	132 ± 17	4.52
**Diastolic BP, mmHg**	74 ± 11	74 ± 11	3.82
**BP < 130/80 mmHg**	225 (41)	190 (40)	3.48
**BP < 140/90 mmHg**	379 (69)	346 (72)	5.54
**CHF**	178 (33)	166 (35)	4.05
**DM**			
Type 1	12 (2)	14 (3)	4.52
Type 2	534 (98)	467 (97)	4.52
**HTN**	533 (98)	467 (97)	3.30
**CVD**	460 (84)	406 (84)	0.44
**CCI**	8.0 ± 2.5	8.1 ± 2.2	3.63
**ACEi/ARB**	280 (51)	241 (50)	2.36
**SGLT2i**	18 (3)	22 (5)	6.57
**GLP-1 RA**	32 (6)	48 (10)	15.30
**SGLT2i and/or GLP-1 RA**	45 (8)	65 (14)	16.99
**Mod- to High-intensity statin**	306 (56)	265 (55)	1.91
**Number of active meds**	7 ± 4	7 ± 4	2.78
**Cr, mg/dL**	1.7 ± 0.4	1.7 ± 0.3	8.78
**eGFR, mL/min/1.73m^2**	37 ± 9	38 ± 8	13.31
**Serum potassium level, meq/L**	4.5 ± 0.5	4.5 ± 0.4	2.51
**HbA1c Value, %**	7.4 ± 1.5	7.4 ± 1.5	1.94
**HbA1c < 7%**	262 (49)	216 (45)	7.02
**UACR, mg/g**<br>**Median (IQR)**	114 (20, 532)	141 (29, 581)	1.33
**Annual UACR**	352 (65)	337 (70%	11.94
**KFRE-5 year risk %**<br>**Median (IQR)**	4.4 (2.4, 9.9)	4.5 (2.5, 9.9)	7.77
**CKD stages**			
2	3 (0.5)	4 (1)	3.41
3a	84 (15)	84 (17)	5.61
3b	359 (66)	321 (67)	2.08
4	99 (18)	72 (15)	8.52
5	1 (0.2)	0 (0)	6.06
**Albuminuria stage**			

A1	159 (30)	121 (26)	10.75
A2	187 (36)	169 (36)	0.05
A3	177 (34)	183 (39)	10.09
**ADI**	68 ± 22	68 ± 21	1.90
**RUCA**			
Metropolitan	434 (80)	348 (73)	16.78
Rural*	111 (20)	132 (27)	16.78

*Other race includes American Indian, Chinese, Indian (Asian), Other Asian, Not specified, and those who declined to answer

**Rural includes micropolitan and rural/town

Abbreviations: BP, blood pressure; UACR, urine albumin-to-creatinine ratio; HbA1c, hemoglobin A1c; ACEi, angiotensin-converting enzyme inhibitor; ARB, angiotensin II receptor blocker; GLP-1RA, glucagon-like peptide-1 receptor agonist; SGLT2i, sodium–glucose cotransporter-2 inhibitor; SD, standard deviation; eGFR, estimated glomerular filtration rate; PCP, primary care provider; BMI, body mass index; CCI, Charlson comorbidity index; IQR, interquartile range; KFRE, kidney failure risk equation; ADI, area deprivation index; RUCA, rural-urban commuting area

Supplemental Table 3. Baseline characteristics of patients enrolled from control versus K-CHAMP practices for ACEi/ARB cohort (*n*=932)

Variable	Control (*N* = 474)<br><br>Mean ± SD or *n* (%)	K-Champ (*N* = 458)<br><br>Mean ± SD or *n* (%)	Standardized Bias
**Age, years**	72 ± 10	73 ± 10	11.67
**Female**	257 (54)	206 (45)	18.56
**Race**			
Black	61 (13)	40 (9)	13.35
White	405 (85)	414 (90)	15.23
Others*	8 (2)	4 (1)	7.25
**Ethnicity**			
Hispanic	5 (1)	4 (1)	1.92
Non-Hispanic	465 (99)	453 (99)	1.92
**Number PCP visits in last 12 months**	4 ± 2	4 ± 3	10.03
**BMI, kg/m^2**	32.5 ± 7.6	32.4 ± 7.3	1.67
**Systolic BP, mmHg**	134 ± 18	133 ± 17	4.47
**Diastolic BP, mmHg**	75 ± 11	75 ± 11	4.29
**BP < 130/80 mmHg**	171 (36)	175 (38)	4.42
**BP < 140/90 mmHg**	309 (65)	317 (69)	8.58
**CHF**	150 (32)	169 (37)	11.09
**DM**			
Type 1	11 (2)	13 (3)	3.27
Type 2	353 (74)	339 (74)	1.04
**HTN**	465 (98)	446 (97)	4.86
**CVD**	383 (81)	384 (84)	7.98
**CCI**	7.3 ± 3.0	7.4 ± 2.6	2.46
**ACEi/ARB**	225 (47)	218 (48)	0.26
**ACEi/ARB exposure days**	204 ± 173	199 ± 171	3.14
**SGLT2i**	14 (3)	16 (3)	3.06
**GLP-1 RA**	25 (5)	34 (7)	8.82
**SGLT2i and/or GLP-1 RA**	35 (7)	47 (10)	10.16
**Mod- to High-intensity statin**	243 (51)	245 (53)	4.46
**Number of active meds**	6 ± 4	6 ± 4	1.88
**Cr, mg/dL**	1.8 ± 0.4	1.8 ± 0.4	3.82
**eGFR, mL/min/1.73m^2**	37 ± 9	38 ± 9	9.35
**Serum potassium level, meq/L**	4.4 ± 0.5	4.4 ± 0.4	0.24
**HbA1c Value, %**	7.3 ± 1.6	7.3 ± 1.6	3.75
**HbA1c < 7%**	208 (50)	187 (48)	3.62
**UACR, mg/g Median (IQR)**	289 (101, 805)	287 (88, 833)	1.59
**Annual UACR**	304 (64)	305 (67)	5.17
**KFRE-5 year risk % Median (IQR)**	7.0 (3.6, 15.2)	6.2 (3.6, 13.1)	11.27
**CKD stages**			
2	2 (0.4)	5 (1)	7.73
3a	82 (17)	78 (17)	0.71
3b	301 (64)	300 (66)	4.18
4	87 (18)	75 (16)	5.23

5	2 (0.4)	0 (0)	9.21
**Albuminuria stage**			
A1	0 (0)	0(0)	
A2	241 (51)	232 (51)	0.38
A3	233 (49)	226 (49)	0.38
**ADI**	68 ± 23	67 ± 22	3.66
**RUCA**			
Metropolitan	387 (82)	357 (78)	9.22
Rural**	87 (18)	101 (22)	9.22

*Other race includes American Indian, Chinese, Indian (Asian), Other Asian, Not specified, and those who declined to answer

**Rural includes micropolitan and rural/town

Abbreviations: BP, blood pressure; UACR, urine albumin-to-creatinine ratio; HbA1c, hemoglobin A1c; ACEi, angiotensin-converting enzyme inhibitor; ARB, angiotensin II receptor blocker; GLP-1RA, glucagon-like peptide-1 receptor agonist; SGLT2i, sodium–glucose cotransporter-2 inhibitor; SD, standard deviation; eGFR, estimated glomerular filtration rate; PCP, primary care provider; BMI, body mass index; CCI, Charlson comorbidity index; IQR, interquartile range; KFRE, kidney failure risk equation; ADI, area deprivation index; RUCA, rural-urban commuting area

Supplemental Table 4. Baseline characteristics of patients enrolled from control versus K-CHAMP practices for statin cohort (*n*=789)

Variable	Control (*N* = 429)<br><br>Mean ± SD or *n* (%)	K-Champ (*N* = 360)<br><br>Mean ± SD or *n* (%)	Standardized Bias
**Age, years**	67 ± 7	66 ± 7	7.55
**Female**	519 (62)	409 (54)	5.33
**Race**			
Black	51 (12)	35 (9)	6.98
White	368 (86)	322 (89)	11.14
Others*	10 (2)	3 (1)	12.02
**Ethnicity**			
Hispanic	2 (0.5)	2 (1)	1.23
Non-Hispanic	424 (99)	357 (99)	1.23
**Number PCP visits in last 12 months**	4 ± 2	4 ± 3	4.67
**BMI, kg/m²**	33.5 ± 7.8	34.2 ± 8.4	8.87
**Systolic BP, mmHg**	132 ± 18	131 ± 16	8.67
**Diastolic BP, mmHg**	77 ± 11	76 ± 10	3.80
**BP < 130/80 mmHg**	165 (38)	151 (42)	7.11
**BP < 140/90 mmHg**	291 (68)	269 (75)	15.27
**CHF**	130 (30)	115 (32)	3.55
**DM**			
Type 1	10 (2)	13 (4)	7.54
Type 2	298 (69)	243 (67)	4.23
**HTN**	407 (95)	338 (94)	4.27
**CVD**	331 (77)	285 (79)	4.87
**CCI**	7.1 ± 3.1	7.0 ± 2.7	2.30
**ACEi/ARB**	204 (48)	172 (48)	0.45
**SGLT2i**	12 (3)	17 (5)	10.13
**GLP-1 RA**	26 (6)	34 (9)	12.68
**SGLT2i and/or GLP-1 RA**	34 (8)	47 (13)	16.80
**Mod- to High-intensity statin**	233 (54)	190 (53)	3.08
**Mod- to High-intensity statin exposure days**	227 ± 167	197 ± 173	17.94
**Number of active meds**	7 ± 4	7 ± 4	0.56
**Cr, mg/dL**	1.8 ± 0.4	1.8 ± 0.4	0.27
**eGFR, mL/min/1.73m²**	38 ± 9	38 ± 9	2.78
**Serum potassium level, meq/L**	4.4 ± 0.5	4.4 ± 0.4	5.03
**HbA1c Value, %**	7.3 ± 1.7	7.2 ± 1.6	3.80
**HbA1c < 7%**	192 (52)	159 (53)	0.38
**UACR, mg/g Median (IQR)**	141 (19, 677)	89 (15, 547)	3.02
**Annual UACR**	250 (58)	215 (60)	2.94
**KFRE-5 year risk % Median (IQR)**	4.7 (2.2, 11.8)	4.2 (2.2, 10.9)	4.30
**CKD stages**			
2	3 (1)	5 (1)	6.79
3a	77 (18)	64 (18)	0.45
3b	282 (66)	235 (65)	0.96
4	65 (15)	56 (16)	1.12

5	2 (0.5)	0 (0)	9.68
**Albuminuria stage**			
A1	118 (30)	119 (35)	9.99
A2	125 (32)	103 (30)	3.85
A3	150 (38)	121 (35)	6.00
**ADI**	68 ± 22	71 ± 22	10.16
**RUCA**			
Metropolitan	343 (80)	266 (74)	14.90
Rural**	85 (20)	94 (26)	14.90

*Other race includes American Indian, Chinese, Indian (Asian), Other Asian, Not specified, and those who declined to answer

**Rural includes micropolitan and rural/town

Abbreviations: BP, blood pressure; UACR, urine albumin-to-creatinine ratio; HbA1c, hemoglobin A1c; ACEi, angiotensin-converting enzyme inhibitor; ARB, angiotensin II receptor blocker; GLP-1RA, glucagon-like peptide-1 receptor agonist; SGLT2i, sodium–glucose cotransporter-2 inhibitor; SD, standard deviation; eGFR, estimated glomerular filtration rate; PCP, primary care provider; BMI, body mass index; CCI, Charlson comorbidity index; IQR, interquartile range; KFRE, kidney failure risk equation; ADI, area deprivation index; RUCA, rural-urban commuting area

Supplemental Table 5. Baseline characteristics of patients enrolled from control versus K-CHAMP practices for GLP-1 RA cohort (*n*=1001)

Variable	Control (*N* = 534)<br>Mean ± SD or *n* (%)	K-Champ (*N* = 467)<br>Mean ± SD or *n* (%)	Standardized Bias
**Age, years**	73 ± 8	73 ± 9	4.73
**Female**	311 (58)	238 (51)	14.65
**Race**			
Black	53 (10)	40 (9)	4.70
White	472 (88)	421 (90)	5.69
Others*	9 (2)	6 (1)	3.31
**Ethnicity**			
Hispanic	5 (1)	4 (1)	0.88
Non-Hispanic	526 (99)	462 (99)	0.88
**Number PCP visits in last 12 months**	4 ± 2	4 ± 3	11.82
**BMI, kg/m^2**	33.1 ± 7.2	33.7 ± 7.6	7.90
**Systolic BP, mmHg**	132 ± 17	132 ± 17	2.73
**Diastolic BP, mmHg**	74 ± 11	74 ± 11	2.32
**BP < 130/80 mmHg**	220 (41)	182 (39)	4.54
**BP < 140/90 mmHg**	373 (70)	336 (72)	4.62
**CHF**	173 (32)	161 (34)	4.41
**DM**			
Type 1	-	-	
Type 2	534 (100)	467 (100)	
**HTN**	521 (98)	454 (97)	2.19
**CVD**	450 (84)	395 (85)	0.86
**CCI**	8.0 ± 2.4	8.1 ± 2.2	5.56
**ACEi/ARB**	276 (52)	234 (50)	3.16
**SGLT2i**	18 (3)	22 (5)	6.81
**GLP-1 RA**	32 (6)	48 (10)	15.73
**GLP-1 RA exposure days**	30 ± 96	32 ± 96	1.40
**SGLT2i and/or GLP-1 RA**	45 (8)	65 (14)	17.50
**SGLT2i and/or GLP-1 RA exposure days**	39 ± 106	43 ± 110	4.49
**Mod- to High-intensity statin**	298 (56)	257 (55)	1.56
**Number of active meds**	7 ± 4	7 ± 4	2.06
**Cr, mg/dL**	1.7 ± 0.4	1.7 ± 0.3	9.33
**eGFR, mL/min/1.73m^2**	37 ± 9	38 ± 8	13.09
**Serum potassium level, meq/L**	4.5 ± 0.5	4.5 ± 0.4	3.17
**HbA1c Value, %**	7.3 ± 1.7	7.2 ± 1.6	0.10
**HbA1c < 7%**	260 (50)	211 (46)	7.91
**UACR, mg/g Median (IQR)**	110 (19, 526)	129 (28, 556)	0.14
**Annual UACR**	347 (65)	329 (70)	11.72
**KFRE-5 year risk % Median (IQR)**	4.3 (2.3, 9.7)	4.5 (2.5, 9.5)	9.35
**CKD stages**			
2	3 (1)	4 (1)	3.51
3a	81 (15)	81 (17)	5.90

3b	353 (66)	312 (67)	1.49
4	96 (18)	70 (15)	8.06
5	1 (0.2)	0 (0)	6.13
**Albuminuria stage**			
A1	158 (31)	120 (26)	10.59
A2	184 (36)	166 (36)	0.33
A3	169 (33)	173 (38)	9.67
**ADI**	68 ± 22	68 ± 21	1.43
**RUCA**			
Metropolitan	424 (79)	337 (72)	16.98
Rural**	109 (21)	129 (28)	16.98

*Other race includes American Indian, Chinese, Indian (Asian), Other Asian, Not specified, and those who declined to answer

**Rural includes micropolitan and rural/town

Abbreviations: BP, blood pressure; UACR, urine albumin-to-creatinine ratio; HbA1c, hemoglobin A1c; ACEi, angiotensin-converting enzyme inhibitor; ARB, angiotensin II receptor blocker; GLP-1RA, glucagon-like peptide-1 receptor agonist; SGLT2i, sodium–glucose cotransporter-2 inhibitor; SD, standard deviation; eGFR, estimated glomerular filtration rate; PCP, primary care provider; BMI, body mass index; CCI, Charlson comorbidity index; IQR, interquartile range; KFRE, kidney failure risk equation; ADI, area deprivation index; RUCA, rural-urban commuting area

Supplemental Table 6. Baseline characteristics of patients enrolled from control versus K-CHAMP practices for SGLT2i cohort (*n*=834)

Variable	Control (*N* = 437)<br><br>Mean ± SD or *n* (%)	K-Champ (*N* = 397)<br><br>Mean ± SD or *n* (%)	Standardized Bias
**Age, years**	73 ± 8	73 ± 9	5.79
**Female**	238 (54)	192 (48)	12.23
**Race**			
Black	29 (7)	27 (7)	0.66
White	399 (91)	366 (92)	3.22
Others*	9 (2)	4 (1)	8.57
**Ethnicity**			
Hispanic	4 (1)	3 (1)	1.80
Non-Hispanic	430 (99)	393 (99)	1.80
**Number PCP visits in last 12 months**	4 ± 2	4 ± 2	7.41
**BMI, kg/m^2**	33.1 ± 7.2	33.8 ± 7.5	9.30
**Systolic BP, mmHg**	132 ± 17	132 ± 16	1.58
**Diastolic BP, mmHg**	74 ± 10	74 ± 11	1.88
**BP < 130/80 mm Hg**	184 (42)	152 (38)	7.79
**BP < 140/90 mmHg**	307 (70)	284 (72)	2.83
**CHF**	135 (31)	125 (32)	1.28
**DM**			
Type 1	-	-	
Type 2	437 (100)	397 (100)	
**HTN**	427 (98)	387 (97)	1.51
**CVD**	374 (86)	331 (83)	6.10
**CCI**	7.9 ± 2.4	8.0 ± 2.2	
**ACEi/ARB**	242 (55)	209 (53)	5.49
**SGLT2i**	18 (4)	20 (5)	4.40
**SGLT2i exposure days**	14 ± 64	16 ± 68	2.83
**GLP-1 RA**	28 (6)	43 (11)	15.81
**SGLT2i and/or GLP-1 RA**	41 (9)	59 (15)	16.85
**Mod- to High-intensity statin**	252 (58)	219 (55)	5.05
**Number of active meds**	7 ± 4	7 ± 3	6.93
**Cr, mg/dL**	1.6 ± 0.3	1.6 ± 0.2	2.54
**eGFR, mL/min/1.73m^2**	40 ± 6.4	40 ± 7	5.83
**Serum potassium level, meq/L**	4.5 ± 0.4	4.5 ± 0.4	0.26
**HbA1c Value, %**	7.4 ± 1.5	7.4 ± 1.5	5.72
**HbA1c < 7%**	201 (47)	175 (44)	5.11
**UACR, mg/g Median (IQR)**	106 (18, 486)	143 (28, 556)	4.52
**Annual UACR**	299 (68)	287 (72)	8.48
**KFRE-5 year risk % Median (IQR)**	3.5 (2.0, 6.9)	3.9 (2.2, 7.3)	5.51
**CKD stages**			
2	3 (1)	4 (1)	3.50
3a	81 (18)	81 (20)	4.72
3b	353 (81)	312 (79)	5.44
4	-	-	

5	-	-	
**Albuminuria stage**			
A1	136 (32)	102 (26)	12.90
A2	153 (36)	136 (35)	2.36
A3	136 (32)	152 (39)	14.62
**ADI**	67 ± 22	68 ± 21	2.52
**RUCA**			
Metropolitan	345 (79)	285 (72)	17.12
Rural**	91 (21)	112 (28)	17.12

*Other race includes American Indian, Chinese, Indian (Asian), Other Asian, Not specified, and those who declined to answer

**Rural includes micropolitan and rural/town

Abbreviations: BP, blood pressure; UACR, urine albumin-to-creatinine ratio; HbA1c, hemoglobin A1c; ACEi, angiotensin-converting enzyme inhibitor; ARB, angiotensin II receptor blocker; GLP-1RA, glucagon-like peptide-1 receptor agonist; SGLT2i, sodium–glucose cotransporter-2 inhibitor; SD, standard deviation; eGFR, estimated glomerular filtration rate; PCP, primary care provider; BMI, body mass index; CCI, Charlson comorbidity index; IQR, interquartile range; KFRE, kidney failure risk equation; ADI, area deprivation index; RUCA, rural-urban commuting area

Supplemental Table7. Number of participants with missing baseline variables

**Outcome variable for the cohort**	**BP, UACR**	**HbA1c**	**ACEi/ARB**	**Statin**	**SGLT2i**	**GLP-1 RA**
**Variable**	**Overall**<br>*N* missing (%)	**Diabetes (Type 1 and 2)**<br>*N* missing (%)	**UACR > 300 mg/g; Diabetes & UACR 30-300 mg/g; hypertension & UACR 30-300 mg/g**<br>*N* missing (%)	**Age 40-75 years**<br>*N* missing (%)	**Type 2 diabetes & eGFR >= 30 mL/min/1.73m^2**<br>*N* missing (%)	**Type 2 diabetes**<br>*N* missing (%)
**Number of patients in the cohort**	***N* = 1596**	***N* = 1027**	***N* = 932**	***N* = 789**	***N* = 834**	***N* = 1001**
**Number of PCP practices in the cohort**	**98**	**97**	**94**	**95**	**95**	**97**
**Ethnicity**	8 (0.50)	4 (0.39)	5 (0.54)	4 (0.51)	4 (0.48)	4 (0.40)
**BMI**	33 (2)	20 (2)	23 (2)	20 (3)	12 (1)	20 (2)
**HbA1c**	333 (21)*	15 (1)	126 (14)*	121 (15)*	12 (1)	15 (2)
**UACR**	145 (9)	31 (3)	-	53 (7)	19 (2)	31 (3)
**KFRE**	145 (9)	31 (3)	-	53 (7)	19 (2)	31 (3)
**Albuminuria stage**	145 (9)	31 (3)	-	53 (7)	19 (2)	31 (3)
**ADI**	47 (3)	33 (3)	29 (3)	34 (4)	30 (4)	31 (3)
**RUCA**	2 (0.13)	2 (0.19)	-	1 (0.13)	1 (0.12)	2 (0.20)

*Cohort included non-diabetic patients

Abbreviations: BP, blood pressure; UACR, urine albumin-to-creatinine ratio; HbA1c, hemoglobin A1c; ACEi, angiotensin-converting enzyme inhibitor; ARB, angiotensin II receptor blocker; GLP-1RA, glucagon-like peptide-1 receptor agonist; SGLT2i, sodium–glucose cotransporter-2 inhibitor; BMI, body mass index; KFRE, kidney failure risk equation; ADI, area deprivation index; RUCA, rural-urban commuting area

Supplemental Table 8. Number of Medication Orders with Missing Dates by Arm

**Medications**	**Total**	**Control**	**K-CHAMP**
ACEi/ARB	257/1900 = 13.5%	130/969 = 13.4%	127/931 = 13.6%
Mod- or High-intensity Statin	207/1815 = 11.4%	106/984 = 10.8%	101/831 = 12.2%
SGLT2i	54/385 = 14.0%	16/160 = 10%	38/225 = 16.9%
GLP-1 RA	55/585 = 9.4%	18/230 = 7.8%	37/355 = 10.4%

Supplemental Table 9. Proportion of patients prescribed guideline-concordant medications in intervention versus usual care arm by year

**Year**	**K-CHAMP** % (95% CI)	**Usual Care** % (95% CI)	**Rate ratio** (95% CI)	**P-value**
ACEi/ARB				
2019	58 (47, 69)	57 (43, 70)	1.02 (0.70,1.33)	>0.9
2020	63 (58, 69)	56 (50, 62)	1.14 (0.98, 1.30)	0.07
2021	61 (57, 66)	57 (52, 61)	1.08 (0.96, 1.20)	0.2
2022	55 (49, 60)	50 (45, 55)	1.09 (0.94, 1.23)	0.2
Mod- or High-intensity Statin				
2019	64 (49, 79)	51 (35, 68)	1.25 (0.75, 1.75)	0.3
2020	63 (56, 70)	67 (60, 73)	0.94 (0.80, 1.08)	0.4
2021	70 (65, 75)	66 (61, 70)	1.07 (0.96, 1.17)	0.2
2022	66 (61, 71)	61 (56, 66)	1.09 (0.97, 1.22)	0.1
SGLT2i				
2019	7 (0, 14)	7 (0, 16)	0.98 (0, 2.69)	>0.9
2020	13 (9, 18)	7 (4, 11)	1.89 (0.66, 3.13)	0.03
2021	25 (20, 29)	10 (7, 13)	2.36 (1.48, 3.24)	<0.001
2022	27 (23, 32)	18 (14, 22)	1.55 (1.11, 1.99)	0.002
GLP-1 RA				
2019	3 (0, 7)	4 (0, 10)	0.74 (0, 2.17)	0.8
2020	14 (9, 18)	6 (3, 8)	2.37 (0.83, 3.91)	0.002
2021	26 (22, 30)	13 (10, 16)	2.02 (1.42, 2.61)	<0.001
2022	28 (24, 33)	1 (12, 18)	1.9 (1.35, 2.44)	<0.001
SGLT2i or GLP-1 RA				
2019	7 (1, 14)	6 (0, 13)	1.23 (0, 2.99)	0.8
2020	22 (17, 28)	10 (7, 14)	2.16 (1.17, 3.14)	<0.001
2021	41 (36, 45)	19 (16, 23)	2.10 (1.63, 2.57)	<0.001
2022	44 (39, 49)	27 (23, 31)	1.63 (1.32, 1.94)	<0.001

Supplemental Figure 1. Effect of K-CHAMP intervention on BP control < 130/80 mmHg compared to usual care control by subgroup

BP < 130/80 mmHg	Usual Care (%) Estimate (SE)	K-CHAMP (%) Estimate (SE)	Relative Benefit (95% CI)	Interaction P-value
**Age**				**0.41**
< 65	28 (4)	46 (5)	1.56 (1.04, 2.07)	
>= 65	42 (2)	45 (2)	1.08 (0.94, 1.22)	
**Gender**				**0.39**
Female	39 (2)	45 (3)	1.17 (0.98, 1.35)	
Male	40 (3)	45 (3)	1.10 (0.90, 1.30)	
**Race**				**0.36**
Others	28 (5)	26 (6)	0.85 (0.38, 1.32)	
White	40 (2)	46 (2)	1.16 (1.01, 1.31)	
**RUCA**				**0.27**
Metropolitan	41 (2)	44 (2)	1.08 (0.94, 1.23)	
Rural/Micropolitan	33 (4)	46 (4)	1.36 (1.01, 1.72)	
**ADI**				**0.36**
< median	43 (3)	47 (3)	1.12 (0.94, 1.30)	
>= median	36 (2)	43 (3)	1.16 (0.95, 1.37)	
**CKD Stages**				**0.42**
3	41 (2)	44 (2)	1.11 (0.96, 1.25)	
4/5	33 (4)	47 (4)	1.33 (0.93, 1.73)	

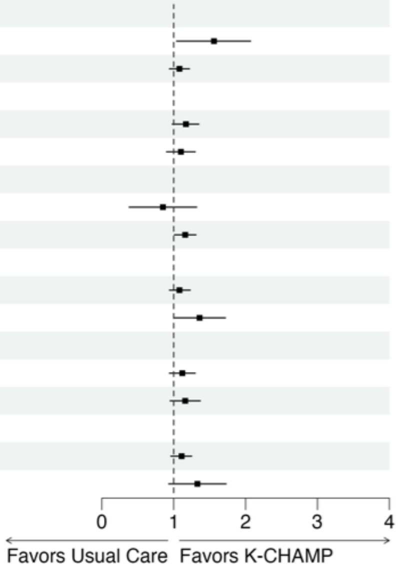

Estimates and confidence intervals are model-based.

Supplemental Figure 2. Effect of K-CHAMP intervention on BP control < 140/90 mmHg compared to usual care control by subgroup

BP < 140/90 mmHg	Usual Care (%) Estimate (SE)	K-CHAMP (%) Estimate (SE)	Relative Benefit (95% CI)	Interaction P-value
**Age**				**0.81**
< 65	67 (4)	67 (4)	0.94 (0.77, 1.10)	
>= 65	72 (2)	77 (2)	1.07 (1.00, 1.14)	
**Gender**				**0.80**
Female	71 (2)	76 (2)	1.06 (0.97, 1.15)	
Male	71 (3)	74 (2)	1.03 (0.93, 1.13)	
**Race**				**0.82**
Others	57 (5)	56 (6)	0.97 (0.69, 1.25)	
White	73 (2)	77 (2)	1.06 (0.98, 1.13)	
**RUCA**				**0.89**
Metropolitan	72 (2)	73 (2)	1.00 (0.93, 1.08)	
Rural/Micropolitan	67 (4)	82 (3)	1.20 (1.04, 1.35)	
**ADI**				**0.80**
< median	74 (2)	75 (2)	1.03 (0.94, 1.12)	
>= median	68 (2)	75 (2)	1.07 (0.97, 1.17)	
**CKD Stages**				**0.75**
3	73 (2)	75 (2)	1.03 (0.96, 1.10)	
4/5	61 (4)	76 (4)	1.15 (0.96, 1.33)	

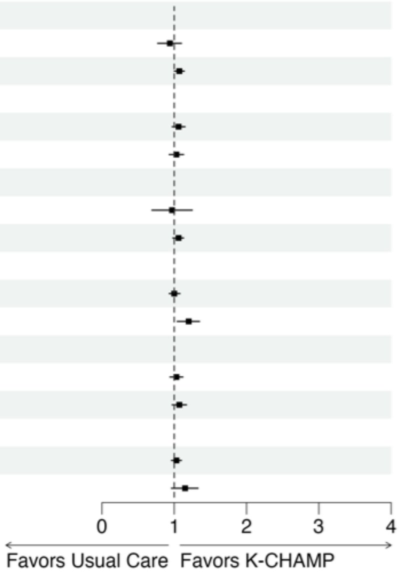

Estimates and confidence intervals are model-based.

Supplemental Figure 3. Effect of K-CHAMP intervention on annual albuminuria testing compared to usual care control by subgroup

Annual UACR	Usual Care (%) Estimate (SE)	K-CHAMP (%) Estimate (SE)	Relative Benefit (95% CI)	Interaction P-value
**Age**				**0.31**
< 65	50 (5)	62 (5)	1.25 (0.94, 1.56)	
>= 65	43 (2)	48 (3)	1.11 (0.94, 1.28)	
**Gender**				**0.27**
Female	42 (3)	51 (3)	1.20 (1.00, 1.41)	
Male	48 (3)	50 (3)	1.05 (0.86, 1.24)	
**Race**				**0.70**
Others	47 (6)	59 (8)	1.20 (0.76, 1.65)	
White	44 (2)	50 (3)	1.13 (0.96, 1.29)	
**RUCA**				**0.55**
Metropolitan	46 (2)	53 (3)	1.16 (1.00, 1.33)	
Rural/Micropolitan	40 (5)	43 (5)	1.07 (0.72, 1.42)	
**ADI**				**0.70**
< median	45 (3)	51 (3)	1.11 (0.92, 1.30)	
>= median	43 (3)	50 (3)	1.16 (0.95, 1.38)	
**CKD Stages**				**0.27**
3	44 (2)	52 (3)	1.17 (1.00, 1.34)	
4/5	45 (5)	44 (5)	0.99 (0.69, 1.28)	

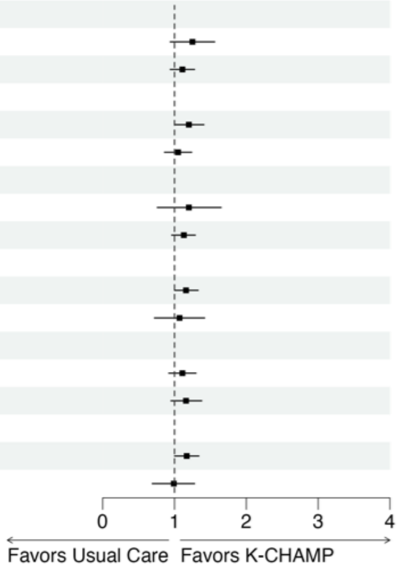

Estimates and confidence intervals are model-based.

Supplemental Figure 4. Effect of K-CHAMP intervention on glycemic control compared to usual care control by subgroup

HbA1c < 7%	Usual Care (%) Estimate (SE)	K-CHAMP (%) Estimate (SE)	Relative Benefit (95% CI)	Interaction P-value
**Age**				**0.92**
< 65	39 (4)	51 (5)	1.29 (0.92, 1.65)	
>= 65	54 (2)	55 (2)	1.04 (0.93, 1.16)	
**Gender**				**0.97**
Female	52 (3)	52 (3)	1.03 (0.88, 1.17)	
Male	51 (3)	56 (3)	1.14 (0.96, 1.32)	
**Race**				**1.00**
Others	51 (6)	47 (6)	0.98 (0.63, 1.32)	
White	51 (2)	55 (2)	1.09 (0.97, 1.21)	
**RUCA**				**0.95**
Metropolitan	50 (2)	53 (2)	1.08 (0.95, 1.21)	
Rural/Micropolitan	56 (4)	57 (4)	1.06 (0.84, 1.28)	
**ADI**				**0.99**
< median	52 (3)	58 (3)	1.11 (0.95, 1.27)	
>= median	51 (3)	50 (3)	1.05 (0.89, 1.20)	
**CKD Stages**				**1.00**
3	49 (2)	54 (2)	1.10 (0.97, 1.23)	
4/5	62 (5)	55 (5)	0.99 (0.74, 1.23)	

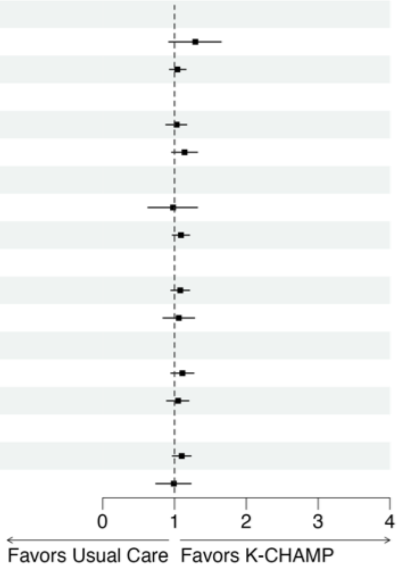

Estimates and confidence intervals are model-based.

Supplemental Figure 5. Effect of K-CHAMP intervention on ACEi/ARB exposure days compared to usual care control by subgroup

ACEi or ARB	Usual Care (days/year) Estimate (SE)	K-CHAMP (days/year) Estimate (SE)	Relative Benefit (95% CI)	Interaction P-value
**Age**				**0.07**
< 65	163 (19)	269 (32)	1.65 (1.06, 2.23)	
>= 65	167 (12)	196 (15)	1.18 (0.90, 1.45)	
**Gender**				**0.28**
Female	175 (14)	205 (19)	1.17 (0.86, 1.48)	
Male	156 (14)	214 (17)	1.38 (1.01, 1.74)	
**Race**				**0.96**
Others	190 (25)	244 (43)	1.28 (0.68, 1.88)	
White	162 (12)	206 (15)	1.27 (0.98, 1.56)	
**RUCA**				**0.53**
Metropolitan	169 (12)	221 (17)	1.31 (1.00, 1.62)	
Rural/Micropolitan	153 (22)	170 (23)	1.11 (0.59, 1.64)	
**ADI**				**0.15**
< median	159 (15)	231 (19)	1.45 (1.06, 1.85)	
>= median	165 (13)	191 (17)	1.16 (0.85, 1.46)	
**CKD Stages**				**0.06**
3	178 (12)	241 (17)	1.35 (1.05, 1.65)	
4/5	92 (14)	79 (14)	0.86 (0.45, 1.27)	

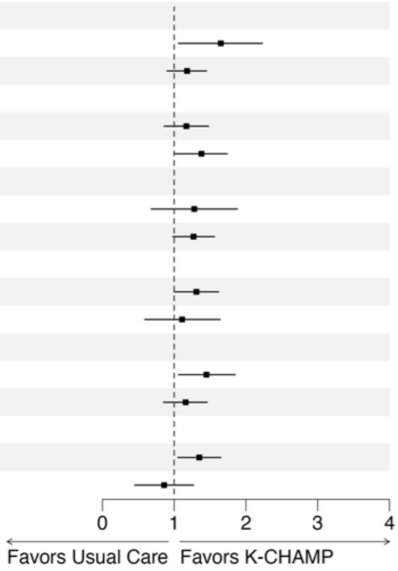

Estimates and confidence intervals are model-based.

Supplemental Figure 6. Effect of K-CHAMP intervention on moderate-high intensity statin exposure days compared to usual care control by subgroup

Mod- or High-intensity Statin	Usual Care (days/year) Estimate (SE)	K-CHAMP (days/year) Estimate (SE)	Relative Benefit (95% CI)	Interaction P-value
**Age**				**0.23**
< 65	235 (19)	247 (24)	1.05 (0.78, 1.32)	
>= 65	187 (12)	234 (17)	1.26 (1.00, 1.51)	
**Gender**				**0.30**
Female	208 (14)	230 (18)	1.10 (0.87, 1.34)	
Male	196 (15)	251 (21)	1.28 (0.98, 1.57)	
**Race**				**0.47**
Others	244 (28)	250 (42)	1.03 (0.60, 1.46)	
White	197 (12)	236 (15)	1.20 (0.99, 1.42)	
**RUCA**				**0.66**
Metropolitan	206 (12)	247 (18)	1.20 (0.97, 1.43)	
Rural/Micropolitan	193 (24)	211 (24)	1.09 (0.69, 1.49)	
**ADI**				**0.30**
< median	187 (14)	240 (21)	1.28 (0.98, 1.59)	
>= median	218 (16)	239 (19)	1.10 (0.85, 1.34)	
**CKD Stages**				**0.92**
3	205 (12)	243 (17)	1.18 (0.96, 1.40)	
4/5	189 (24)	219 (29)	1.16 (0.73, 1.59)	

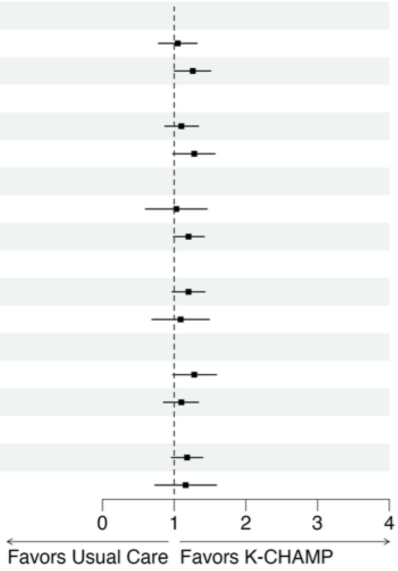

Estimates and confidence intervals are model-based.

Supplemental Figure 7. Effect of K-CHAMP intervention on SGLT2i exposure days compared to usual care control by subgroup

SGLT-2i	Usual Care (days/year) Estimate (SE)	K-CHAMP (days/year) Estimate (SE)	Relative Benefit (95% CI)	Interaction P-value
**Age**				**0.47**
< 65	47 (13)	65 (16)	1.39 (0.48, 2.30)	
>= 65	30 (6)	53 (9)	1.80 (1.13, 2.48)	
**Gender**				**0.05**
Female	22 (5)	53 (11)	2.37 (1.25, 3.49)	
Male	45 (9)	59 (11)	1.30 (0.74, 1.87)	
**Race**				**0.04**
Others	56 (18)	36 (16)	0.64 (0.00, 1.29)	
White	30 (6)	57 (10)	1.90 (1.24, 2.57)	
**RUCA**				**0.82**
Metropolitan	33 (6)	57 (10)	1.75 (1.08, 2.41)	
Rural/Micropolitan	32 (11)	51 (12)	1.59 (0.39, 2.78)	
**ADI**				**0.95**
< median	36 (7)	61 (12)	1.72 (0.94, 2.50)	
>= median	30 (7)	50 (10)	1.69 (0.88, 2.49)	

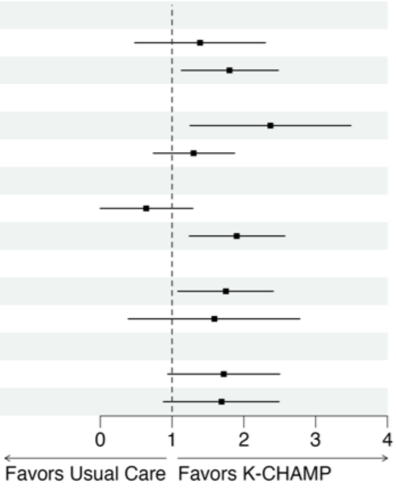

Estimates and confidence intervals are model-based.

Supplemental Figure 8. Effect of K-CHAMP intervention on GLP-1RA exposure days compared to usual care control by subgroup

GLP-1 RA	Usual Care (days/year) Estimate (SE)	K-CHAMP (days/year) Estimate (SE)	Relative Benefit (95% CI)	Interaction P-value
**Age**				**0.85**
< 65	53 (13)	146 (29)	2.75 (1.03, 4.48)	
>= 65	24 (4)	63 (10)	2.58 (1.45, 3.72)	
**Gender**				**0.80**
Female	30 (6)	77 (13)	2.56 (1.35, 3.77)	
Male	29 (6)	79 (14)	2.76 (1.33, 4.19)	
**Race**				**0.07**
Others	57 (16)	70 (23)	1.23 (0.11, 2.36)	
White	26 (4)	77 (12)	2.93 (1.71, 4.15)	
**RUCA**				**0.51**
Metropolitan	28 (5)	79 (13)	2.81 (1.57, 4.04)	
Rural/Micropolitan	36 (10)	73 (16)	2.05 (0.33, 3.78)	
**ADI**				**0.18**
< median	22 (5)	42 (9)	1.89 (0.87, 2.92)	
>= median	35 (7)	102 (16)	2.89 (1.55, 4.22)	
**CKD Stages**				**0.29**
3	33 (5)	82 (12)	2.48 (1.47, 3.50)	
4/5	12 (5)	54 (16)	4.34 (0.00, 8.72)	

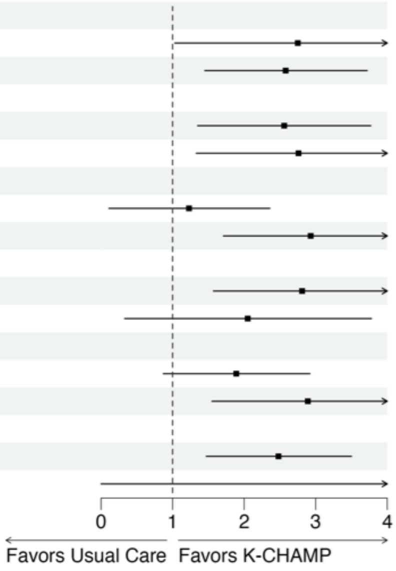

Estimates and confidence intervals are model-based.

Supplemental Figure 9. Effect of K-CHAMP intervention on SGLT2i and/or GLP-1RA exposure days compared to usual care control by subgroup

SGLT-2i and/or GLP-1 RA	Usual Care (days/year) Estimate (SE)	K-CHAMP (days/year) Estimate (SE)	Relative Benefit (95% CI)	Interaction P-value
**Age**				**0.55**
< 65	77 (15)	192 (32)	2.49 (1.23, 3.74)	
>= 65	45 (6)	96 (12)	2.12 (1.43, 2.80)	
**Gender**				**0.41**
Female	42 (6)	101 (13)	2.44 (1.54, 3.34)	
Male	62 (9)	127 (17)	2.03 (1.27, 2.79)	
**Race**				**0.04**
Others	82 (18)	92 (25)	1.11 (0.32, 1.91)	
White	47 (6)	114 (13)	2.44 (1.68, 3.20)	
**RUCA**				**0.44**
Metropolitan	50 (6)	117 (14)	2.35 (1.57, 3.13)	
Rural/Micropolitan	58 (14)	104 (16)	1.79 (0.67, 2.91)	
**ADI**				**0.67**
< median	46 (7)	95 (14)	2.04 (1.23, 2.85)	
>= median	56 (8)	126 (16)	2.26 (1.41, 3.11)	
**CKD Stages**				**0.55**
3	58 (7)	125 (14)	2.16 (1.52, 2.80)	
4/5	16 (5)	45 (11)	2.78 (0.54, 5.01)	

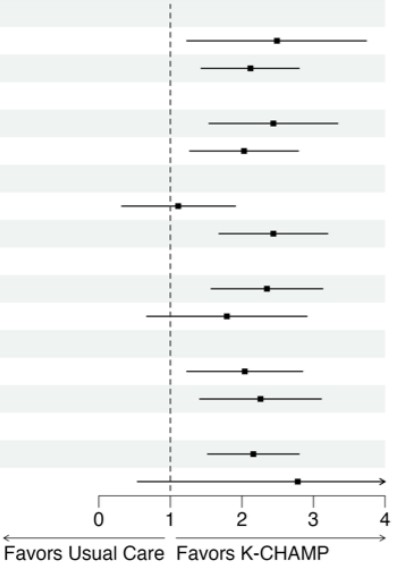

Estimates and confidence intervals are model-based.

Supplemental Figure 10. Sensitivity analyses results: 30 days imputation for prescription orders with missing end dates

Process Measures	N	Usual Care (%) Estimate (SE)	K-CHAMP (%) Estimate (SE)	Relative Benefit (95% CI)	P-values	FDR-adjusted P-values
BP < 130/80 mmHg	1596	39 (2)	45 (2)	1.15 (1.00, 1.31)	0.05	0.11
BP < 140/90 mmHg	1596	71 (2)	75 (2)	1.06 (0.98, 1.14)	0.14	0.16
Annual UACR	1596	45 (2)	51 (2)	1.14 (0.98, 1.29)	0.09	0.14
HbA1c < 7%	1027	52 (2)	54 (2)	1.10 (0.93, 1.27)	0.23	0.23
**Medication**	**N**	**Usual Care (days/year) Estimate (SE)**	**K-CHAMP (days/year) Estimate (SE)**	**Relative Benefit (95% CI)**	**P-values**	**FDR-adjusted P-values**
ACEi or ARB	932	113 (8)	142 (11)	1.26 (0.99, 1.54)	0.06	0.11
Mod- or High-intensity Statin	789	148 (9)	178 (13)	1.20 (0.95, 1.45)	0.12	0.15
SGLT-2i	834	22 (4)	37 (6)	1.69 (1.14, 2.23)	0.01	0.04
GLP-1 RA	1001	23 (4)	57 (9)	2.51 (1.51, 3.51)	0.003	0.01
SGLT-2i and/or GLP-1 RA	1001	39 (5)	84 (10)	2.14 (1.48, 2.80)	<0.001	0.006

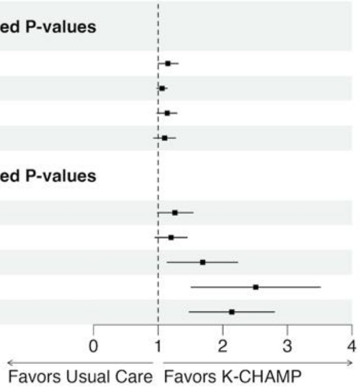

Supplemental Figure 11. Sensitivity analyses results: 90 days imputation for prescription orders with missing end dates

Process Measures	N	Usual Care (%) Estimate (SE)	K-CHAMP (%) Estimate (SE)	Relative Benefit (95% CI)	P-values	FDR-adjusted P-values
BP < 130/80 mmHg	1596	39 (2)	45 (2)	1.15 (1.00, 1.31)	0.05	0.10
BP < 140/90 mmHg	1596	71 (2)	75 (2)	1.06 (0.98, 1.14)	0.14	0.16
Annual UACR	1596	45 (2)	51 (2)	1.14 (0.98, 1.29)	0.09	0.14
HbA1c < 7%	1027	52 (2)	54 (2)	1.10 (0.93, 1.27)	0.23	0.23
**Medication**	**N**	**Usual Care (days/year) Estimate (SE)**	**K-CHAMP (days/year) Estimate (SE)**	**Relative Benefit (95% CI)**	**P-values**	**FDR-adjusted P-values**
ACEi or ARB	932	122 (9)	155 (11)	1.27 (0.99, 1.55)	0.06	0.10
Mod- or High-intensity Statin	789	158 (9)	188 (13)	1.18 (0.95, 1.42)	0.12	0.15
SGLT-2i	834	24 (4)	41 (7)	1.70 (1.14, 2.26)	0.01	0.04
GLP-1 RA	1001	24 (4)	61 (9)	2.57 (1.54, 3.59)	0.003	0.01
SGLT-2i and/or GLP-1 RA	1001	41 (5)	90 (10)	2.18 (1.51, 2.86)	<0.001	0.005

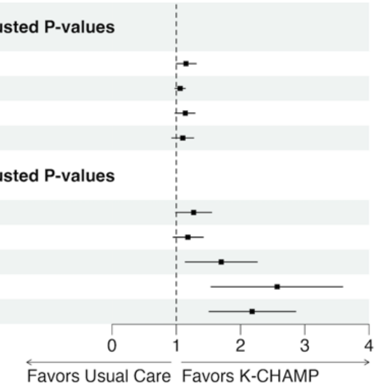

Supplemental Figure 12. Sensitivity analyses results: Patient-specific median duration imputation for prescription orders with missing end dates*

Process Measures	N	Usual Care (%) Estimate (SE)	K-CHAMP (%) Estimate (SE)	Relative Benefit (95% CI)	P-values	FDR-adjusted P-values
BP < 130/80 mmHg	1596	39 (2)	45 (2)	1.15 (1.00, 1.31)	0.05	0.10
BP < 140/90 mmHg	1596	71 (2)	75 (2)	1.06 (0.98, 1.14)	0.14	0.18
Annual UACR	1596	45 (2)	51 (2)	1.14 (0.98, 1.29)	0.09	0.14
HbA1c < 7%	1027	52 (2)	54 (2)	1.10 (0.93, 1.27)	0.23	0.23
**Medication**	**N**	**Usual Care (days/year) Estimate (SE)**	**K-CHAMP (days/year) Estimate (SE)**	**Relative Benefit (95% CI)**	**P-values**	**FDR-adjusted P-values**
ACEi or ARB	932	114 (9)	157 (13)	1.37 (1.02, 1.72)	0.04	0.09
Mod- or High-intensity Statin	789	159 (10)	188 (15)	1.18 (0.91, 1.45)	0.19	0.21
SGLT-2i	834	24 (5)	40 (7)	1.68 (1.16, 2.21)	0.01	0.03
GLP-1 RA	1001	24 (4)	58 (9)	2.39 (1.44, 3.35)	0.004	0.02
SGLT-2i and/or GLP-1 RA	1001	42 (5)	86 (10)	2.08 (1.45, 2.70)	<0.001	0.007

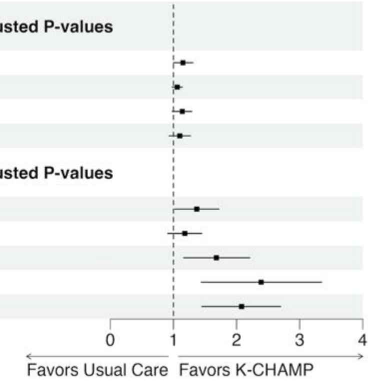

** Missing medication end dates were imputed based on the median duration of the patient's prior medication orders for the same medication; if the patient had no other non-missing medication order available, the median duration across similar prescription orders with non-missing end-dates from other patients were used*
